# An integration-to-bound model of decision-making that accounts for the spectral properties of neural data

**DOI:** 10.1038/s41598-019-44197-0

**Published:** 2019-06-10

**Authors:** Ramón Guevara Erra, Marco Arbotto, Aaron Schurger

**Affiliations:** 1 Integrative Neuroscience and Cognition Center, UMR 8002, Université Paris Descartes, Paris, France; 2 Integrative Neuroscience and Cognition Center, UMR 8002, CNRS, Paris, France; 3 Master of Biomedical Engineering,Université Paris Descartes, Paris, France; 4 INSERM, Cognitive Neuroimaging Unit, Gif sur Yvette, 91191 France; 5 Commissariat à l’Energie Atomique, Direction des Sciences du Vivant, I2BM, NeuroSpin center, Gif sur Yvette, 91191 France; 6 Department of Psychology, Crean College of Health and Behavioral Sciences, Chapman University, Orange, CA, USA; 7 Institute for Interdisciplinary Brain and Behavioral Sciences, Chapman University, Irvine, CA, USA

**Keywords:** Decision, Dynamical systems, Neural encoding

## Abstract

Integration-to-bound models are among the most widely used models of perceptual decision-making due to their simplicity and power in accounting for behavioral and neurophysiological data. They involve temporal integration over an input signal (“evidence”) plus Gaussian white noise. However, brain data shows that noise in the brain is long-term correlated, with a spectral density of the form 1/f^α^ (with typically 1 < α < 2), also known as pink noise or ‘1/f’ noise. Surprisingly, the adequacy of the spectral properties of drift-diffusion models to electrophysiological data has received little attention in the literature. Here we propose a model of accumulation of evidence for decision-making that takes into consideration the spectral properties of brain signals. We develop a generalization of the leaky stochastic accumulator model using a Langevin equation whose non-linear noise term allows for varying levels of autocorrelation in the time course of the decision variable. We derive this equation directly from magnetoencephalographic data recorded while subjects performed a spontaneous movement-initiation task. We then propose a nonlinear model of accumulation of evidence that accounts for the ‘1/f’ spectral properties of brain signals, and the observed variability in the power spectral properties of brain signals. Furthermore, our model outperforms the standard drift-diffusion model at approximating the empirical waiting time distribution.

## Introduction

Neurophysiological recordings and behavioral data support the view that decision-making in humans is intrinsically a noisy process. This is clearly demonstrated in the most popular model of decision-making, the drift-diffusion model (DDM). The DDM has, over three decades or so, accounted for a large amount of experimental evidence - behavioral and more recently electrophysiological^1–10^. An internal random variable called ‘evidence’ quantifies the amount of information in favor of a given decision. Accumulated evidence evolves in time during the decision process, until it reaches a threshold and a decision is made (integration to bound). Mathematically, the DDM is a stochastic differential equation for the accumulated evidence (z), with a drift (deterministic) term, quantifying the rate of accumulation of evidence, and a diffusion (stochastic) term. We consider a more general version of the DDM currently used in the literature, by adding leakage to the drift term. This model is usually referred as a “leaky stochastic accumulator model”^7^ (LSA, also known as the Ornstein-Uhlenbeck process^11^). It is given by the stochastic differential equation,

1 $$\frac{dz}{dt}=az+b+{\Gamma }(t),$$

where *a* and *b* are constant coefficients, and *Γ*(*t*) is additive Gaussian white noise. The distribution of reaction times and other behavioral variables, as well as their neurophysiological correlates have been modeled using this approach. The typical assumption in the literature is that there is a constant drift rate and (possibly also) leak in the deterministic part of the stochastic differential equation (SDE), and that the diffusion part is given by constant white noise. Equation (1) can be seen as a particular case of a more general SDE, the Langevin equation, widely used in physics, chemistry and biology to model stochastic processes^11,12^. The Langevin equation contains two additive terms, a deterministic *g*(*z*, *t*) and a stochastic *h*(*z*, *t*) term, which are functions of *z* and *t* (in the following, we will refer to them as deterministic and stochastic functions),

2 $$\frac{dz}{dt}=g(z,t)+h(z,t){\Gamma }(t)={D}^{(1)}(z,t)+\sqrt{{D}^{(2)}(z,t)}{\Gamma }(t),$$

where we have also expressed the equation in terms of *D*^(1)^(*z*, *t*) and *D*^(2)^(*z*, *t*), which are, respectively, the drift and diffusion coefficients of the corresponding Fokker-Planck equation^12^. The LSA (equation (1)) is a Langevin equation with *g*(*z*) = *az* + *b* and h(*z*) = 1.

Within the context of behavioral experiments, the LSA fits the data well. However, the possibility that a more general Langevin process (equation (2)) is taking place during decision making remains an open question: as recent work indicates, neurophysiological measures may also reflect the process of accumulation of evidence^1,6,9^ and we hypothesize that the corresponding stochastic process may differ from the standard diffusion model. Therefore, in this study we set out to use neurophysiological data to check for possible generalizations of the standard diffusion model, by attempting to reconstruct the underlying stochastic process directly from the data.

The reconstruction of dynamical systems has a long history within the framework of nonlinear dynamics^13^. Recently, data-based reconstruction has been extended to noisy systems, in particular, to a large class of dynamical systems (the so-called Markovian systems, characterized by the lack of memory effects) that can be modeled by the Langevin equation^14,15^. In a neurophysiological context, this method has been successfully applied to EEG data to derive the underlying brain dynamics during epileptic seizures^16^. Interestingly, the derived Langevin equation strongly differs from drift-diffusion models of decision-making. In particular, the diffusion coefficient has a nonlinear dependence on voltage for both within- and between-seizure periods. This is an important fact, that points towards the possible presence of nonlinearities in the Langevin coefficients both in abnormal brain dynamics and in normal subjects, and emphasizes the general importance of the specific nonlinear dependencies of Langevin coefficients for discriminating physiological states. If these nonlinearities were present during the accumulation of evidence, then an important revision of current accumulator models might be called for, especially in contexts where the drift term is quite small relative to the noise, as in the case of spontaneous self-initiated movement^7,9,17^.

A second important aspect of neurophysiological data is that its spectral properties seem to be connected to brain function. Recent investigations into the nature of background pink noise (or ‘1/f’ noise, with spectral density given by a power law 1/f^α^) in the central nervous system have begun to reveal that the degree of autocorrelation in resting-state brain activity may be crucial for brain function^18^. The spectral properties of this background noise change in different task contexts and with different states of vigilance (for example during sleep). The standard diffusion model, having a constant diffusion coefficient, cannot generate noise with variable 1/f exponent. Indeed, its output has fixed spectral density of 1/f  ^2^ (Brownian noise), whereas pink noise has 1/f^α^ with α in the approximate range 0 < α < 2. If the character of the noise itself matters to neuronal computation and ensuing behavior, then allowing flexible autocorrelation SDE models can lead to novel and powerful predictions that can be formally tested. With the surge of interest in so-called “resting-state” brain activity and 1/f noise in the brain, attention may begin to shift towards modeling the noise more faithfully (the diffusion process) and not just the drift coefficient.

Here we derive diffusion models of decision-making from the underlying neural activity. We also develop a generalization of the leaky stochastic accumulator model using a Langevin equation whose non-linear stochastic term allows for trajectories with varying levels of temporal autocorrelation, referred as ‘nonlinear model’, because it is nonlinear both in the deterministic and the stochastic part of the corresponding Langevin equation. Both the LSA and the nonlinear model are deduced using a data-driven approach, whereby we reconstruct the coefficients of the Langevin equation from experimental data. We applied this approach to magnetoencephalographic (MEG) data recorded while subjects performed a spontaneous movement task^19^. This kind of task has recently been modeled using a leaky stochastic accumulator, with the “evidence” (drift term) being very small^7,9^, representing an inclination to possibly initiate movement sometime in the near future. On the basis of the reconstructed SDE we built behavioral models through an optimization procedure, in such a way that the models’ output approach as much as possible the empirical waiting time distributions. The nonlinear model represents the simplest diffusion model that accounts for accumulation of evidence while staying true to the spectral properties of brain signals. In addition, this nonlinear model better approximates the empirical waiting time distribution, as compared to the LSA.

## Results

### Magnetoencephalographic data modeled with a Langevin equation

**Figure 1:**
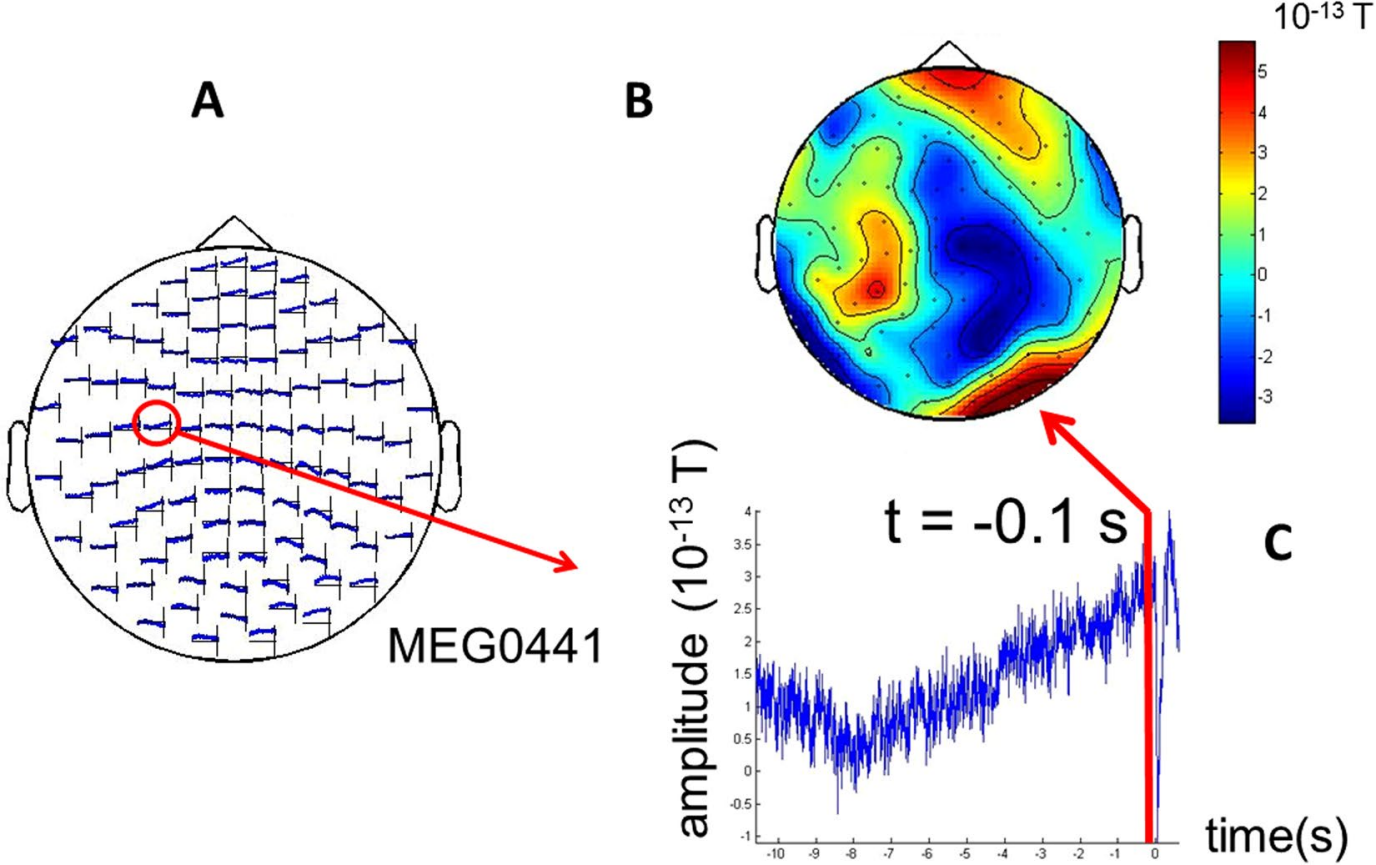
Evoked responses and topography. Evoked responses (event-related fields or ERFs) at magnetometers from a representative subject (subject 5). Magnetic fields are measured in Tesla (T) and typically in the f  T (10^−15^ T) range, and time is measured in seconds. (**A**) Multiple plot, (**B**) Topography at a latency of 0.1 seconds before the button press (subject response) and (**C**) Evoked response at a representative channel: MEG0441.

The magnetoencephalography (MEG) data recorded while subjects performed spontaneous self-initiated movements (see Methods) reveal a characteristic accumulation process, typical of decision making paradigms (in the following we denote the MEG signals from magnetometers as *x*, measured in units of femto Tesla [f  T = 10^−15^ T]; since MEG is reference free and no specific baseline was applied, *x* represent relative values). This consisted of activity that slowly grows with time towards a saturation point, and then returns to baseline. This can be seen in Fig. 1, showing MEG magnetometer’s signals averaged over trials and presented as a topographic map over the surface of the head. The slowly developing averaged fields were mostly present over centro/parietal sensors, and can be associated with the late positive evoked potential, previously observed in electroencephalography (EEG) experiments on decision making^7^. One important goal was to analyze not the averaged activity, but individual trial activity. We treated individual-trial MEG signals as outcomes of a stochastic processes, which, on average, give rise to slow-wave fields showing accumulation of evidence. In other words, we assume, as in a large part of the literature, that the process of accumulation of evidence correlates with slowly changing potentials. But we concentrate on individual trials to acquire information about the underlying stochastic process, considering that any averaging procedure might mask this relevant information.

As explained in detail in the Methods section, we followed a fitting procedure assuming that the underlying stochastic process can be described by a Langevin equation. It is possible to fit the Langevin equation (by fitting we mean calculating its deterministic and stochastic parts), to the first and second moments of the input data. This data-driven approach was applied to two types of data sets: Case 1: data sets consisting of concatenated trial traces (the input data in the algorithm is a vector that contains all of the available trials, see Methods section for the definition of a trial). Case 2: the traces from individual trials themselves (the input for the algorithm is the vector of all data samples of a single trial, see Methods section). The logic behind this two-tiered approach was that the algorithm performs better for a larger amount of data (Case 1, with an input vector consisting of all the data samples available, that is, all the available trials), whereas individual trial traces should better reflect the underlying stochastic process (there are changes in the underlying dynamics from trial to trial, so Case 2 should better represent the dynamics of individual trials). The results were the following.

#### Case 1: concatenated trials (input data: all trials)

**Figure 2:**
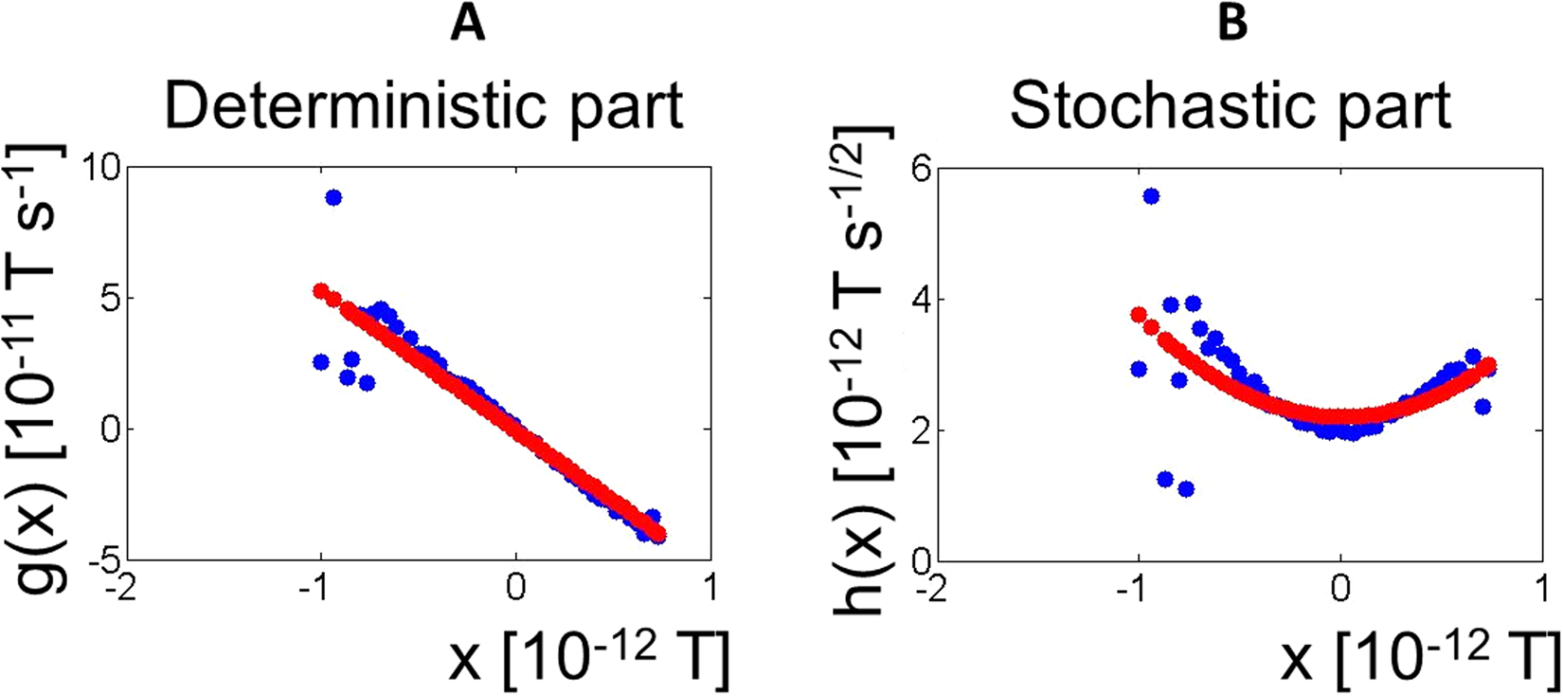
Single channel reconstructed Langevin functions using all available trials (Case 1). Underlying dynamics fitted to a Langevin equation: $\frac{{\rm{dx}}}{{\rm{dt}}}=g(x)+h(x){\rm{\Gamma }}(t)$, where *x* is the amplitude of the magnetic field measured by the magnetometers, as in Fig. 1. *g*(*x*) and *h*(*x*) (Langevin functions) are the deterministic and stochastic parts of the Langevin equation, respectively. Γ(t) is Gaussian white noise. The input data was a vector of all available trials taken together (Case 1 in the Results section). (**A**) *g*(*x*) and (**B**) *h*(*x*) for a representative subject at a representative channel (subject 5, channel MEG0441). The best fit is: *g*(*x*) = *mx* + *n* and *h*(*x*) = *px*^2^ + *qx* + *r*. Blue: data. Red: best fit. Similar figures for all subjects and channels are available in the Supplementary Material.

In Fig. 2 we show a typical example of the output of the fitting algorithm for case 1. The left-side graph depicts the deterministic part of the Langevin equation (function *g*(*x*) in the Langevin equation, equal to the drift coefficient in the corresponding Fokker-Planck equation^12^) and as can be seen, it is possible to fit it to a straight line, *g*(*x*, *t*) = *mx* + *n*.Here, the X axis is *x*, the magnetic flux through individual SQUIDs, measured in the standard units (T = Tesla, and amplitude of magnetic flux have values typically in the fT range [fT = 10^−15^ T]) used in MEG experiments and the Y-axis is the function *g*(*x*) as in equation (2), calculated using equations (6) and (7) (Methods section). The right-side graph is the stochastic part of the Langevin equation (function *h*(*x*)), the square root of the diffusion coefficient in the corresponding Fokker-Planck equation that was best fit by a parabola, *h*(*x*, *t*) = *px*^2^ + *qx* + *r*.

This result can be summarized in the following equation,

3 $$\frac{dx}{dt}=mx+n+(p{x}^{2}+qx+r){\Gamma }(t),$$

**Figure 3:**
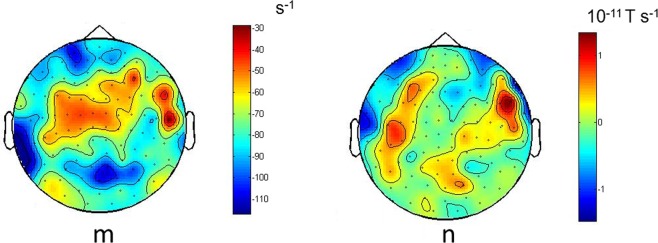
Topographies of the reconstructed Langevin function *g*(*x*) using all available trials (Case 1). The Langevin functions are defined as in Fig. 2. Topographies of coefficients *m* and *n* in *g*(*x*) = *mx* + *n* (obtained from the reconstructed Langevin function *g*(*x*) using all trials (Case 1) for a representative subject (subject 5)). Similar figures for all subjects and channels are available in the Supplementary Material.

**Figure 4:**
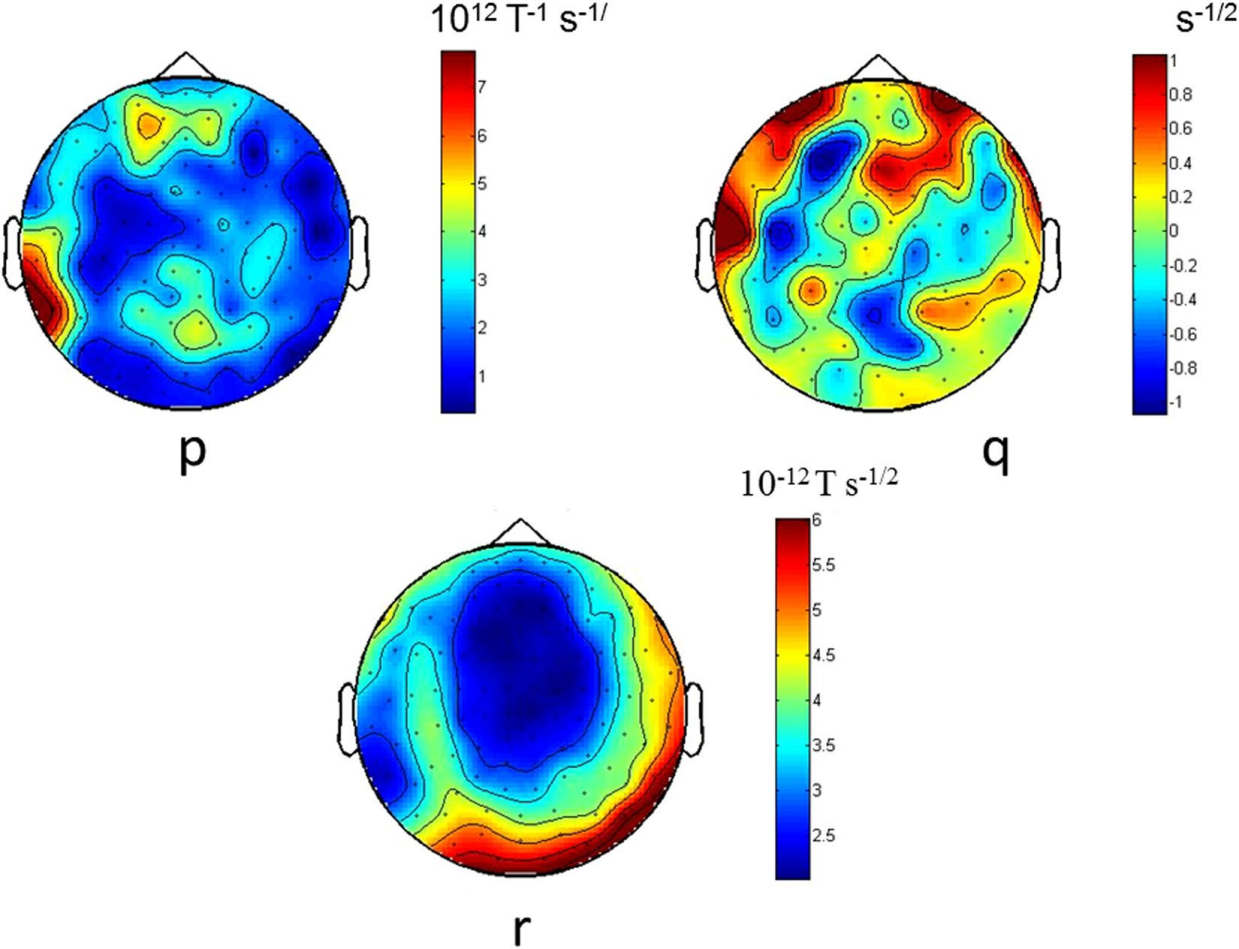
Topographies of the reconstructed Langevin function *h*(*x*) using all available trials (Case 1). Topographies of coefficients *p*, *q* and *r* in *h*(*x*) = *px*^*2*^ + *qx* + *r* (all trials subject 5, as in Fig. 3). Similar figures for all subjects and channels are available in the Supplementary Material.

There is variation of the coefficients *m*, *n*, *p*, *q*, *r* across channels. This can be observed in the topographic maps of magnetometer signals shown in Figs 3 and 4 for *g*(*x*) and *h*(*x*) respectively.

#### Case 2: individual traces (input data: one trial)

**Figure 5:**
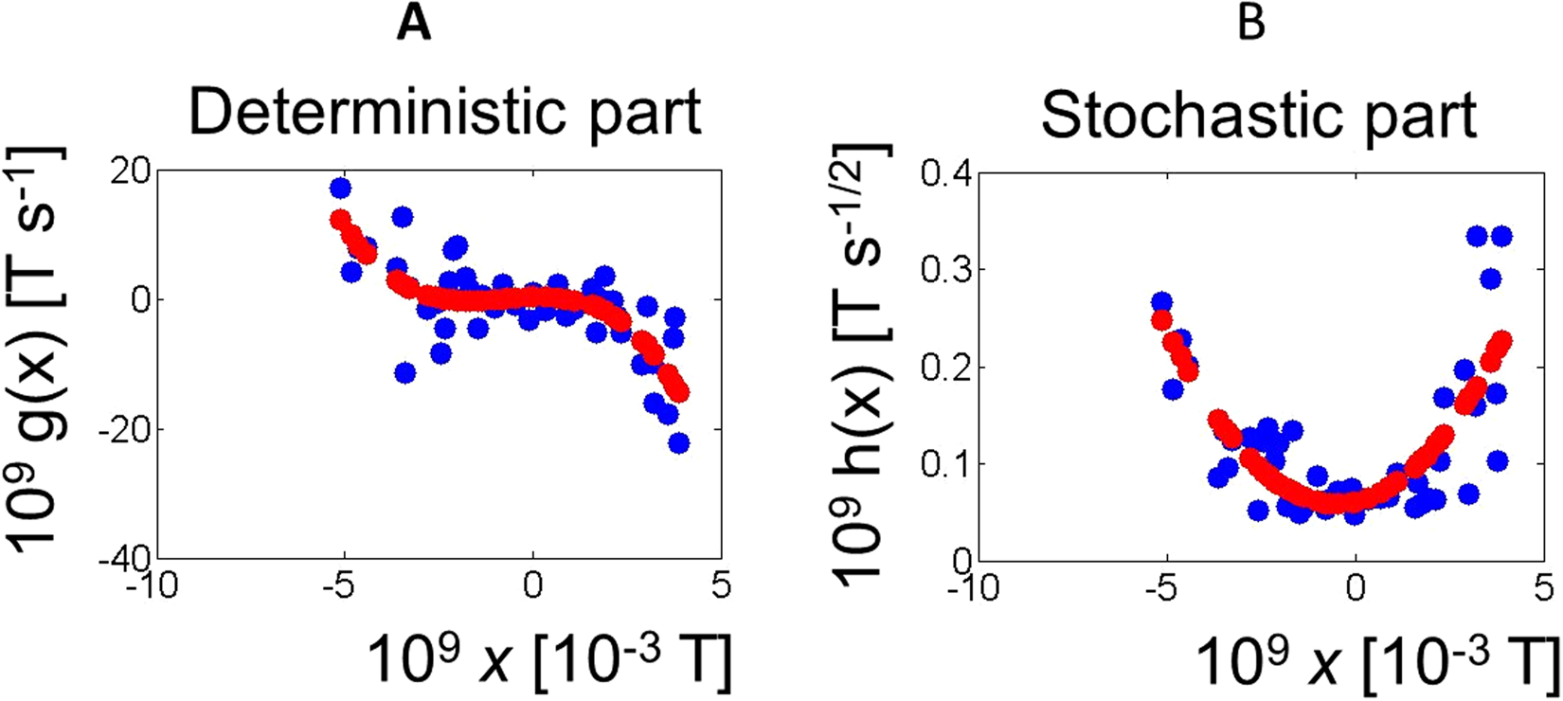
Single channel reconstructed Langevin functions for one trial (Case 2). Single trial (Case 2) reconstructed Langevin functions *g*(*x*) and *h*(*x*) (defined as in Fig. 2) for a representative subject, at a representative channel (subject 5, channel MEG0441). (**A**) Deterministic part, with best fit *g*(*x*) = *ax*^3^ + *bx*^2^ + *cx* + *d*. (**B**) Stochastic part, with best fit *h*(*x*) = *Ax*^2^ + *Bx* + *C*. Blue: data. Red: best fit. For numerical reasons (see Methods section) and for visualization purposes, *x*, *g*(*x*) and *h*(*x*) where scaled by a factor of 10^9^. Similar figures for all subjects (at all channels) are available in the Supplementary Material.

The Langevin functions of single trials do not follow the same functional dependency on *x* as in the case above. Indeed, the best fit for the deterministic part of individual trial traces is not linear but cubic (Fig. 5), in equations,

4 $$\frac{dx}{dt}=(a{x}^{3}+b{x}^{2}+cx+d)+(A{x}^{2}+Bx+C){\Gamma }(t),$$

whereas the stochastic function is still quadratic. It is useful to compare the functional form of the deterministic parts of the Langevin equation in both (3) and (4). It is observed that individual trials are well fitted to cubic functions, and the coefficients of the fit are different for every trial. We conclude that this is the best fit to the underlying dynamics represented by MEG signals observed in centro/parietal regions.

**Figure 6:**
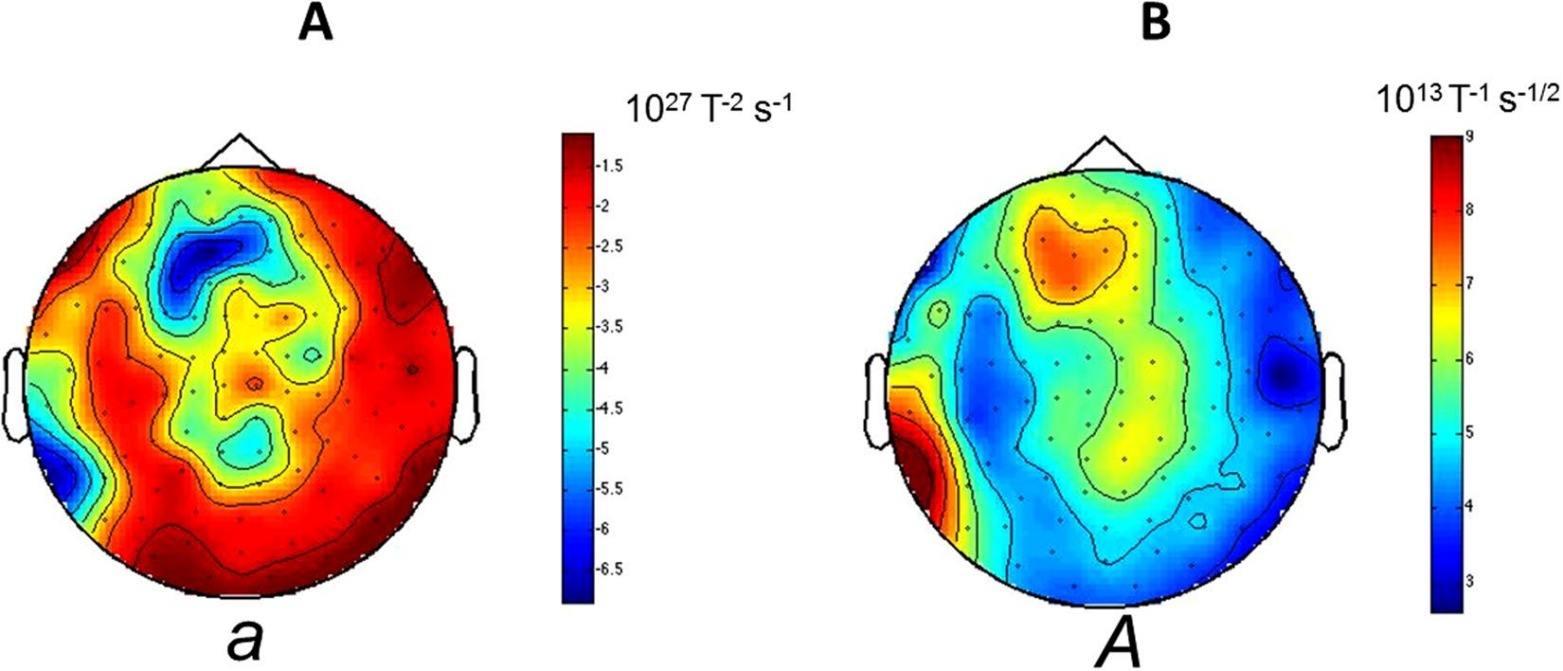
Topographies of the trial - averaged reconstructed Langevin functions (Case 2). The coefficients in the Langevin equation (*a*, *A*, etc) were calculated on each single trial, as in Fig. 5. The average was taken across trials, to obtain trial-averaged coefficients (note that this procedure is different to the one used in Figs 2, 3 and 4, where the coefficients are obtained by using all trials taken together). Topographies of the trial-averaged Langevin coefficients *a* and *A* for a representative subject (subject 5). For simplicity, only the leading terms (*a* and *A*) in the equations for *g*(*x*) and *h*(*x*) are depicted, since the other terms are much smaller (see Supplementary Materialfor the other coefficients topographies). (**A**) Topography of coefficient *a* in *g*(*x*) = *ax*^3^ + *bx*^2^ + *cx* + *d*, (**B**) Topography of coefficient *A* in *h*(*x*) = *Ax*^2^ + *Bx* + *C*. Similar figures for all subjects are available in the Supplementary Material.

The coefficients *a*, *b*, *c*, *d* and *A*, *B*, *C* in equation (4) vary across trials and channel location. This is illustrated in Fig. 6, where trial-averages of the coefficients were represented topographically on scalp maps. The trial-averaged topographies were calculated as follows: the coefficients (*a*, *b*, etc) were calculated for each trial at each channel. Subsequently, trial averages of the coefficients were taken at each channel. Note that, in contrast to Case 1, here the trial average was taken over the fitted coefficients (each coefficient fitted on a single trial, whereas in Case 1 each coefficient was fitted by using all trials). Variability across channels can easily be observed from those topographic maps (Fig. 6). In Fig. 6, only the leading coefficients in the polynomial (*a* for *g*(*x*) and *A* for *h*(*x*)) are presented.

### Models of accumulation of evidence derived from magnetoencephalographic data

In this section we build models of accumulation of evidence derived from magnetoencephalographic (MEG) data on the basis of the results obtained in the previous section.

**Figure 7:**
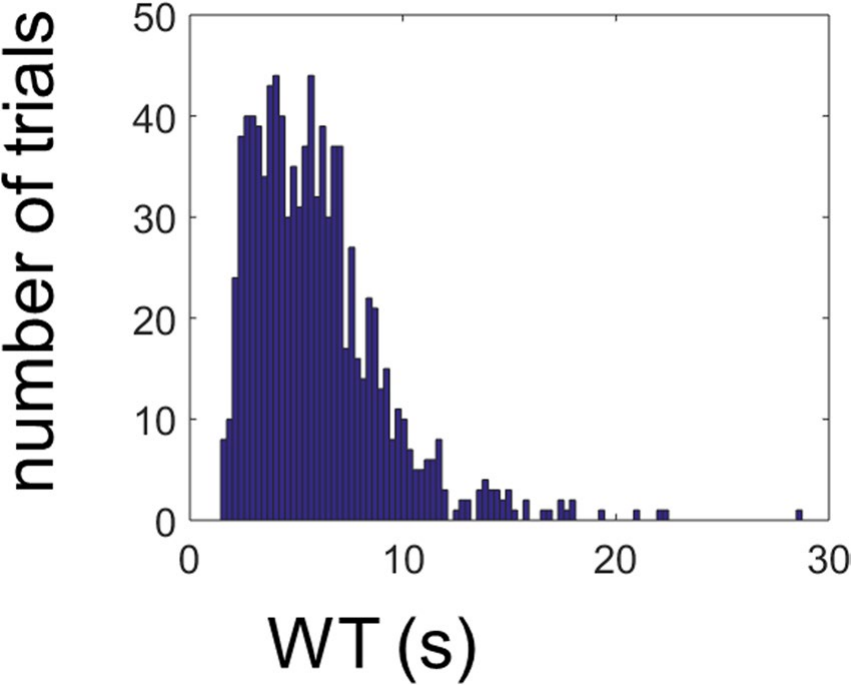
Waiting time distribution of the Libet experiment. Waiting time distribution (histogram) from the empirical data across all the data set (15 subjects).

Our approach is the following: we construct the models in such a way that they approach, as much as possible, the waiting time distribution observed in the experiment (Fig. 7). The waiting time (WT) is defined as the time from the beginning of a given trial to the button press (see Methods). Each model consists of a stochastic differential equation (Langevin equation) and a threshold. The magnitude of the threshold is given by the parameter *β*. Once the Langevin equation is derived from MEG data and the threshold is chosen, a numerical simulation of the model gives a distribution of waiting times. For the numerical simulations, waiting times are defined as the time elapsed from the beginning of the simulation until the threshold is crossed. Two types of models were considered: a linear model (LSA) given by Langevin equation (3) and a nonlinear model given by Langevin equation (4). This can be written in compact form as,

5 $$\frac{dz}{dt}={P}_{G}(z)+{P}_{H}(z){\Gamma }(t),$$

where *P*_*G*_(*z*) and *P*_*H*_(*z*) are polynomials of degrees *G* and *H*, respectively, defining the deterministic and stochastic parts of the Langevin equation in terms of the accumulated evidence *z*. The degree of the polynomials are *G* = 1 and *H* = 0 for the LSA and *G* = 3 and *H* = 2 for the nonlinear model.

The parameters of the Langevin equation are derived from MEG data, in a similar fashion as in the previous section (*Case* 2), but with some differences, as explained in the Methods. In particular, the Langevin coefficients were unique for a single channel, in contrast with *Case* 2 above, where the parameters of the Langevin equation were different from trial to trial. In other words, for a given subject and channel, we found a single Langevin equation. For each value of the threshold, a different WT distribution was obtained, so an optimization was done in order to find the value of threshold crossing at which the waiting time distribution was as close as possible to the WT distribution of the whole behavioral data set (that is, to the WT distribution for all trials and subjects, see Fig. 7). Quantitatively, this was achieved by calculating the Euclidean distance *E*(*β*) between distributions (see Methods). The minimum of this function determined the optimal value of the threshold (*β*_*o*_). Finally, a single best model is found for which *E*(*β*) is minimal across subjects and channels, both for LSA and the nonlinear Langevin equation.

**Figure 8:**
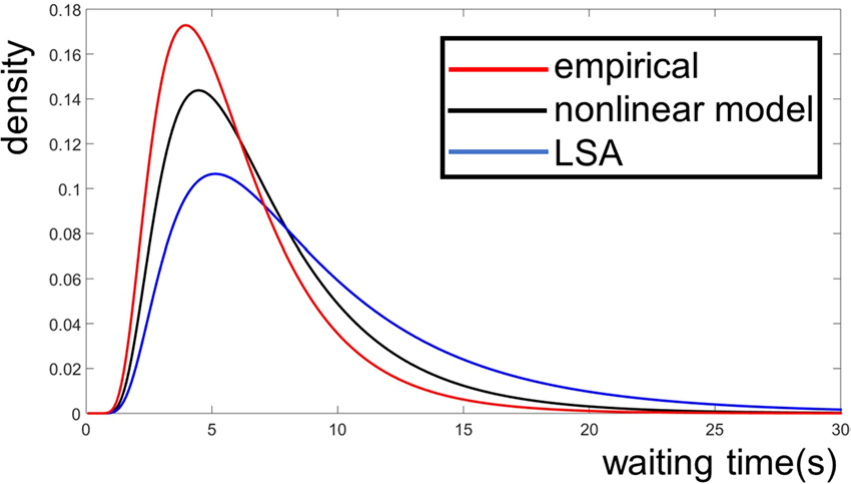
Models of accumulation of evidence: LSA and nonlinear model. Waiting time density function of the LSA, nonlinear model and behavioral data. The waiting times (WT) distributions are fit to an inverse Gaussian function with parameters *μ* (mean) and *λ* (shape parameter). Red: empirical normalized WT distribution (see also Fig. 7) with *μ* = 5.95 and *λ* = 21.96. Blue: optimal LSA across subjects and channels, with equation $\frac{{\rm{dz}}}{{\rm{dt}}}=({\rm{az}}+{\rm{b}})+A{\rm{\Gamma }}(t)$, where *z* is accumulated evidence and *t* time. The parameters of the model are: *a* = −0.29, *b* = 1.43, *A* = 1.78 and threshold crossing *β* = 6.7. The parameters of the LSA WT distribution are *μ* = 9.16 and *λ* = 22.46. Black: optimal nonlinear model across subjects and channels, with equation $\frac{{\rm{dz}}}{{\rm{dt}}}=({{\rm{az}}}^{3}+{{\rm{bz}}}^{2}+{\rm{cz}}+{\rm{d}})+\,({{\rm{Az}}}^{2}+{\rm{Bz}}+{\rm{C}}){\rm{\Gamma }}(t).$ The parameters of the model are: *a* = −0.03, *b* = 0.17, *c* = 0, *d* = 0.81, *A* = 0.01, *B* = −0.01, *C* = 1.72 and *β* = 6.8. The parameters of the nonlinear model WT distribution are *μ* = 7.01 and *λ* = 22.55. The distances of the empirical WT distribution to the LSA and the nonlinear model WT distributions are, D_LSA_ = 0.15 and D_non_ = 0.07, respectively.

The results are shown in Fig. 8. Both the LSA and the nonlinear model produce long tail WT distributions with averages and standard deviations close to the experimental WT distribution. However, the performance of the nonlinear model is better than the LSA in approximating the empirical WT distribution (Fig. 8). Indeed, the distance between the optimal nonlinear model and the empirical WT distributions was *D*_*non*_ ≡ *D*(*ρ*_*non*_, *ρ*_*emp*_) = 0.07, whereas the distance between the optimal LSA and the empirical WT distributions was *D*_*LSA*_ ≡ *D*(*ρ*_*LSA*_, *ρ*_*emp*_) = 0.15 (for the definition of distance between distributions see Methods).

## Discussion

Our main result is the derivation of diffusion models of decision making on the basis of neural data. Specifically, we have derived stochastic differential equations that describe the neural dynamics of human subjects, as recorded by magnetoencephalography (MEG) during a self-initiated movement task^7^. We have found that these equations determine a drift-diffusion process, if the analysis is concentrated on areas where the readiness potential is observed. Using that restricted data set we have derived models of accumulation of evidence whose output approximates the empirical waiting time distribution, suggesting that the corresponding brain areas may be involved, or at least correlate with the process of decision making.

**Figure 9:**
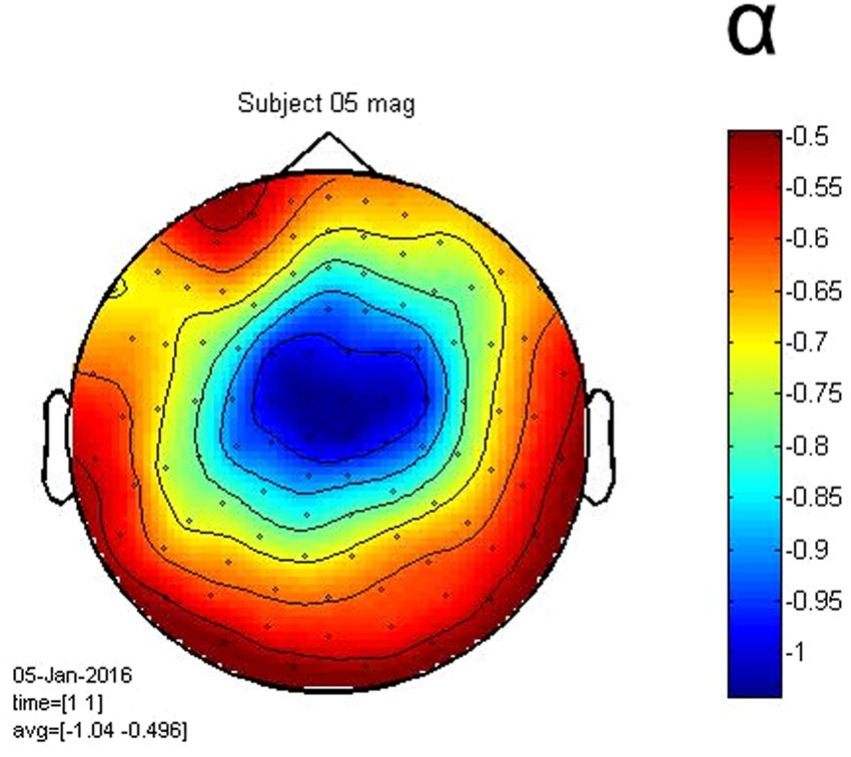
Spectral properties of MEG data. (**A**) Topographic map of the coefficient *α* in *S*~1*/f*^*α*^ power law, obtained from the power spectral density *S* at all channels for subject 5.

In doing so, we have proposed a generalization of the classical diffusion model of decision-making. This generalized model differs from the classical model (LSA) in that it is nonlinear both in the deterministic and in the stochastic part. The output of this generalized model has spectral properties that coincide with those of the electrophysiological signals (by construction), so in this respect differs from the LSA, which only allows for a Brownian process and does not follow the variability of the spectral properties of the underlying signals (the variability in α, the exponent in the spectral density 1/f^α^). In Fig. 9 a topographic map of the coefficient *α* of the spectral density power law 1/f^α^ for one subject shows that *α* is closer to 1 (‘1/f’ power law) in a region where the readiness potential is typically observed. Furthermore, the nonlinear model outperforms the LSA in terms of WT distributions: *D*_*non*_ < *D*_*LSA*_(Fig. 8). However, none of the models fit the empirical WT distribution very well, due to the fact that the variability of the waiting time data for individual participants is too great for appropriate data fitting. For several participants, it is not even possible to achieve any fit (the expected inverse Gaussian or other). The reason is that the number of trials is relatively small for each participant. We would have needed more trials per participant to achieve a good fit. Instead, we had use the whole data set. By contrast, we chose not to concatenate the MEG data across several participants, as the parameters of the models vary considerably across participants. Pooling data across participants would have erased important information about the nonlinearity of the subjacent MEG signals, and as a result would have made comparisons between models impossible. We speculate that fitting group-level behavioral data with subject-level data-driven-models reflected on the empirical waiting time distributions not being perfectly fit by the models.

Regarding equation (4), other functions could have been used to fit the data, but we have attempted to use polynomials with the minimal degree that fit the reconstructed Langevin functions. In the Supplementary Material, the Langevin functions *g*(*x*) and *h*(*x*) are shown for all the subjects, normalized such that all have the same mean value. It can be observed that the normalized functions obtained have the following property: the function *g*(*x*) is approximately odd (*g*(*x*) = −*g*(−*x*)) in *x* for all subjects, channels, and trials, whereas the function *h*(*x*) is approximately even in *x* (*h*(*x*) = *h*(−*x*)). The corresponding best fits for those functions can be achieved with polynomials of odd degree (*n* = 1, 3, 5…) for *g*(*x*) and even degree (*n* = 2, 4, 6…) for *h*(*x*). We have chosen polynomials of minimal order to avoid over-fitting. Indeed, we used a polynomial of order 2 to fit the function *h*(*x*). For the function *g*(*x*) it was clear from the obvious nonlinearity of the traces (see Supplementary Material, where it can be observed that the traces have local maxima and minima) that the case *n* = 1 does not produce a good fit to the data. Therefore, we have chosen *n* = 3, that is a cubic polynomial, for the best fit. However, we emphasize that this is not the only approach to generating signals with a 1/f^α^ spectrum, and we do not claim generality in this context. Our approach is heuristic, given that a fit is never exact, i.e. it is possible to fit data by several models, so, even if we have found a SDE that best fits the data, we do not exclude that other more complex models might also fit the data equally well (using rational, but not integer coefficients for the exponents in the functions *g*(*x*) and *h*(*x*), for example).

The assumption that there is a brain correlate for the decision variable is supported by empirical evidence. Recently, sensory evidence and decision signals in EEG recordings were found in a gradual target detection task in humans, using electroencephalographic recordings^6^. These accumulation-to-bound signals exhibited the same dynamics (and anatomical location in centro-parietal areas) as those associated with decision-making in neurophysiological experiments in monkeys, and were independent of sensory modality. As for self-initiated actions, neural correlates of a decision variable were identified in single neurons in rats that could abort waiting for a large reward and spontaneously opt for a smaller, but immediate reward^9^. Two populations of neurons in the secondary motor cortex (M2) were interpreted as “evidence” (mean firing rate during the trial correlated with waiting time) and decision signals (ramping at rates proportional to waiting times until a threshold was reached). Finally, an association between a decision variable and the electrophysiological signal has been established in^7^, in the very same data set we are using in the current work.

One could argue that the spontaneous initiation of movement does not require the integration of evidence. But this just depends on what is meant by “accumulated evidence”. Even in a task where the subject is given an indefinite amount of time to perform a movement (as in^7^), no subject ever took longer than about 20 seconds to produce a movement, and typically took no longer than 5 to 7 seconds. This holds even when subjects are clearly told and reminded that there is no time limit. In this context, one can consider the demand characteristics of the task as the “evidence”. It is implicit in the nature of the task that subjects should produce a movement within a reasonable amount of time, because the experiment is about making movements. Subjects understand this without it ever being said explicitly and one can argue that it takes the place of sensory evidence in the accumulator model. Indeed, without at least a small amount of drift in the leaky stochastic accumulator, it was not possible to fit the behavioral and brain data from subjects performing the task^7^.

Scale-free dynamics –exhibiting a power spectrum of the type 1/f^α^ – are characteristic of complex systems, and brain activity is not an exception: electrophysiological recordings indicate widespread scale-free dynamics, both in the resting state and during cognitive tasks^18^. Our data also show the power spectrum of 1/f type (Fig. 9). Recent research has emphasized the importance of the broadband (typically 1/f) component of brain dynamics, in contrast to the also pervasive oscillatory (narrowband) activity observed in brain recordings^20^. In a recent review, experimental work from the visual, language and motor systems was revisited, showing that power spectral measurements reveal broadband spectral changes (in scalp EEG recordings) being correlated with cortical function, and tracking the activation of asynchronous local neuronal populations^21^. In agreement with this literature, we have found that the parameters of our nonlinear model change spatially over the electrode array (Fig. 6).

The time series of neural activity in the brain has spectral density of the form 1/f^α^, varying typically in the range 1 < α < 2. α is not constant in real neural data, it varies over time and according to task demands. The canonical accumulator model of decision making, by contrast, generates time series with α = 2, exactly. To the extent that accumulator models are used to model neural time series, arguably the output of the model should be faithful to the spectral properties of those neural time series. This is not possible with a canonical accumulator model (e.g. drift-diffusion model, or leaky stochastic accumulator). Here we have used the Langevin equation to derive a generalization of the leaky stochastic accumulator model, from MEG data, that allows for varying α in the 1/f^α^ power spectrum, and that approximates the empirical WT distribution better than the leaky stochastic accumulator. With increasing interest in 1/f noise in the brain, the focus on computational modeling might begin to shift towards modeling the noise more faithfully (in the diffusion process) and not just the drift coefficient. Our work here represents a first step in that direction.

## Materials and Methods

Simultaneous magnetoencephalography (MEG), electroencephalography (EEG), and behavioral data were collected as part of a previous study, for which details of the EEG and behavioral data have already been reported^7^. Here we focus our analyses on the MEG data from that study.

### Human subjects

A total of 16 subjects participated in the experiment (6 female, mean age 28 years, 1 left handed), all with normal or corrected-to-normal vision. All subjects gave written informed consent and were paid for their participation in the experiment. The research protocol was approved by the Ethics Committee on Human Research at the Commissariat à l’Energie Atomique et aux Energies Alternatives (CEA, DRF/I2BM, NeuroSpin, Gif-sur-Yvette, France) and conformed to the Declaration of Helsinki. Two of the subjects did not exhibit a readiness potential (a negative slope in voltage from −1.00 s to −0.25 s at either Cz, C3, or FC1) and so were excluded from the analyses leaving N = 14. In the original study, each subject completed two different tasks, a “classic” Libet task^19^, and a slight variant of it that we called the “interruptus” task. Here we focus on the data from the classic task.

### Stimuli and task

The task was almost identical to that of^19^. Subjects monitored an analog clock dial on a back-projection screen positioned at a distance of about 60 cm. A small dot made one cycle around the perimeter of the clock every 3 seconds (50 ms per “second” on the clock). The subject held a cylindrical button bar in his/her dominant hand and pressed the button at the top of the bar with his/her thumb. The subject sat comfortably with his/her head inside the MEG helmet, inside of a magnetically and electrically shielded chamber. The subject was instructed to fixate a small ‘+’ sign positioned at the center of the clock dial throughout the duration of each trial.

The appearance of the fixation cross in the center of the screen signaled the beginning of each trial. The dot would then begin to circle the clock face, always starting at the top. The subject was instructed to allow the dot to make one full revolution around the edge of the clock face, and that s/he could then press the thumb button at any time after that. The subject was instructed to be spontaneous: do not pre-plan the time of the button press, but rather make a single abrupt flexion of the thumb at a random instant. Subjects were specifically told to always keep the thumb in contact with the button, such that the movement in question would always be an abrupt flexion of the thumb rather than a lift-drop type of movement. Subjects were reminded that after the dot made one full cycle around the clock face, the button press could happen at any time. No subject ever waited longer than 30 seconds before pressing the button. Each subject completed anywhere from 49 to 99 trials. For the complete details of the task see^7^.

### Magnetoencephalographic (MEG) recordings

MEG data were recorded inside of a magnetically shielded chamber at 1000 Hz with a 333 Hz analog lowpass filter (no high-pass filtering was applied – data were recorded in DC mode). The Elekta NeuroMag ® MEG system has 102 magnetometers and 102 pairs of orthogonal planar gradiometers. The subject also wore a 60-channel MEG-compatible EEG cap. [For details of the EEG data see^7^. Here we have focused on the MEG data primarily due to its higher spatial resolution, and the analyses reported here were carried out only on the MEG data.] Four small coils used to estimate the subject’s head position inside the MEG helmet were affixed to the EEG cap, and their positions digitized with respect to three fiducial points (nasion and just above the tragus on each ear). The subject’s head position was measured at the beginning of each block of trials and this information was used offline during the data analysis to transform the data from each block into a head-centered coordinate frame.

Electro-oculogram (EOG; horizontal and vertical) and electro- myogram (EMG; flexor pollicis longus muscle) were also recorded. Apart from delaying the signals by approximately 50 ms, time locking to the button press yielded similar results to time locking to the rectified high-pass filtered EMG. EMG data were either unavailable or unreliable for four subjects (among the N = 14 subjects), so all time-locking was done with respect to the time of the button press.

### MEG data pre-processing

The first step in preprocessing the MEG data was to apply the MaxFilter software application developed by Elekta NeuroMag. This software tool automatically detects and interpolates “bad” channels and applies the Signal Space Separation (SSS) algorithm which attenuates magnetic field fluctuations originating outside of the MEG helmet. MaxFilter then transforms the data into a head-centered coordinate frame using the head-position measurements taken at the beginning of each block of trials. The data were also downsampled to 250 Hz at this stage. The remainder of the preprocessing was done using the FieldTrip (fieldtrip.fcdonders.nl) toolbox for MatLab ® (MathWorks Inc). Ocular and cardiac artifacts were isolated and removed using independent components analysis^22^, and trials that still had significant artifacts after this step were identified by visual inspection and excluded from the analyses.

### MEG data analyses and statistics

Drift and diffusion coefficients were calculated using a method to extract model equations from noisy experimental data^14^. This method has been validated in the context of electrophysiological signals^15^ and in particular, it has been successfully used to characterize EEG recordings from epileptic patients^16^. Here, we briefly describe this method. The main idea is that noisy dissipative dynamical systems can be fully described by the Langevin equation, equation (2), that is, a first order differential equation with a deterministic (first term in the RHS of equation (2)) and a stochastic function (second term in the RHS of equation (2)). In this equation noise is additive, generated by a Gaussian white noise process *Γ*(*t*), assumed to be uncorrelated, <*Γ*(*t*)*Γ*(*t*′) >= *δ*(*t* − *t*′) and with zero mean, <*Γ*(*t*) >= 0. The functions *g*(*x*, *t*) and *h*(*x*, *t*) are related to the drift (*D*^(1)^(*x*, *t*)) and diffusion (*D*^(2)^(*x*, *t*)) coefficients of the Fokker – Planck equation^11,12^ by the relations *D*^(1)^(*x*, *t*) = *g*(*x*, *y*) and *D*^(2)^(*x*, *t*) = *h*^2^(*x*, *y*).

The stochastic differential equation (2) was used to describe the underlying dynamics generating the MEG time series observed in the experiment. The corresponding stochastic process describes a dynamics lacking memory effects (Markovian)^15^: the time development of the system is completely characterized by its current state, independently of its previous history. This assumption is typically satisfied by electrophysiological brain signals^16^.

It is further assumed that the functions *g*(*x*, *t*) and *h*(*x*, *t*) are not explicitly time-dependent.

With these assumptions, it is possible to calculate the deterministic and the stochastic functions of the Langevin equation (2), using the Itô stochastic calculus^23^,

6 $$g(x)={\mathrm{lim}}_{\tau \to 0}\frac{x(t+\tau )-x(t)}{\tau }={D}^{(1)},$$

7 $${h}^{2}(x)={\mathrm{lim}}_{\tau \to 0}\frac{{(x(t+\tau )-x(t))}^{2}}{\tau }={D}^{(2)}.$$

In other words, drift and diffusion coefficients are defined as the first and second conditional moments of the stochastic variable *x*(*t*). The numerical evaluation of the limits in Equations (6) and (7) was obtained by extrapolation: (1) For a time series *x*(*t*) the range of variation of *x* was calculated:[*x*_*min*_, *x*_*max*_]. (2) That range was divided into 100 bins. For each bin, a set was constructed that contains all the possible values of *x*(*t*_*i*_ + *τ*) such that *x*(*t*_*i*_) = *x*, for a given time step *τ* (with *τ* ranging from 10/*fs* to 1/*fs* (10 values), *fs* being the sampling frequency of 250 Hz). (3) For each *τ*, the averages *A*_1_ =< *x*(*t* + *τ*) − *x*(*t*)> and *A*_2_ = (*x*(*t* + *τ*) − *x*(*t*))^2^ were calculated (of course *A*_1_and *A*_2_ cannot be calculated for the last 10 samples of the time series, so those final samples (“right boundary”) were not used for the analysis). (4) A polynomial function was constructed that best fit the functions *A*_1_(*τ*) and *A*_2_(*τ*). (5) The limit in equations (6) and (7) was found by extrapolating the values of the averages as *τ* → 0 for the functions *A*_1_(*τ*) and *A*_2_(*τ*).

We defined *x*(*t*) as being the MEG time series of an individual trial in the interval where the evoked potential was found. We took as the beginning of this interval the latency of 2.5 seconds prior to button pressing, and as the end of the interval, the latency of 0.5 seconds after button pressing. No further preprocessing was applied on top of the preprocessing described in section “Data Preprocessing”. For the calculation of the Langevin coefficients in Case 1 of the Results section we proceeded as follows: for each subject, at each MEG channel and for each condition, a data set was created with all the trials (ranging from 48 to 99 for different subjects) so as to constitute a vector *y*(*t*) = [*x*_1_(*t*), *x*_2_(*t*), … *x*_*N*_(*t*)], where *x*_*i*_(*t*) is the time series for trial *i* and *N* is the total number of trials. In other words, in Case 1, individual trials (*x*_*i*_(*t*)) are concatenated to constitute a vector (*y*(*t*)) for the analysis (in contrast, in Case 2 data from an individual trial [“individual traces”: *x*_*i*_(*t*))] are used for the analysis). This extended data set contains a large number of samples (each trial contains 750 samples, for a total duration of 3 s). This is very useful for the statistical evaluation of the Langevin coefficients, given that the efficiency of algorithm to fit the Langevin coefficients is proportional to the number of samples in the data. We used the extended data set *y*(*t*) instead of *x*(*t*) in equations (6) and (7), following the five steps described above (for the calculation, the data samples at the “right boundaries” of each trial were not used, see step 3 above). For numerical reasons (to avoid division by very small numbers in the algorithm), it was convenient to re-scale the values of *x* in Case 2. The scale factor used is 10^9^ (see Fig. 5). To summarize, the algorithm applied is the same in both cases (1 and 2) but it is applied to different input: in Case 2 it is applied to individual trials and in Case 1 to a concatenated vector containing all the trials available.

### Behavioral models and simulations

#### Langevin equation

The parameters of the Langevin equation were extracted from neural data in the following way: (1) A set of MEG channels that maximally resemble a readiness potential was chosen for the analysis (a different set for each subject). For each subject, this set was constructed by defining a weight (*W*) characterizing the resemblance of a given channel to a readiness potential. To this end, the event related field ERF(*t*) was calculated as the mean across trials in the usual way. A region of analysis was chosen with a time window of 0.4 s centered at the time of button press (*t* = 0). Within that region, the maximum of the ERF was calculated, and *W* was defined as the temporal average of the ERF around that maximum, in a time window of 0.2 s. *W* is proportional to the ERF maximum value in the vicinity of *t* = 0, as expected for a readiness potential. Evoked responses were visually inspected for each of the channels chosen according to our method and they do indeed correspond to the intuitive idea of a readiness potential, a slow buildup of activity. Finally, we chose a set of 5 channels having the largest values of *W*, for each subject. (2) For each channel in this set, MEG data were first re-scaled and a time window chosen. The scaling factor was taken to be 10^13^, in such a way that the relationship between neural data (*x*) and accumulated evidence (*z*) was *z* = 10^13^ *x*. The scale factor was taken arbitrarily in order to get an evidence variable that was manageable for simulations (numbers of the size of *x*, that is, of the order of 10^12^ can lead to numerical overflow during simulations) and also compatible with the orders of magnitude of *z* typically used in the literature. The time window chosen started at a latency of 3 seconds prior to button pressing and finished at button pressing (−3 s < *t* < 0 s). We have chosen this interval, and not the interval used for the complete MEG analysis exposed in the previous section (that extends to 0.5 s after button pressing), because we are interested in the dynamics related to accumulation of evidence, developing before button pressing. Indeed, we observe a change in dynamics as seen in the ERF at button pressing or slightly after (~0.2 s), resulting in the culmination of the readiness potential and the return to baseline. (3) The parameters of the Langevin equation were calculated from the ensemble of trials. For each trial, *g*(*z*) and *h*(*z*) were calculated as in equations (6) and (7), as explained in the previous section. However, the polynomial fit to these functions was not done trial by trial, as was the case in the MEG data fitting (*Case* 2). Instead, all the trials were used for polynomial fitting, in order to get a single set of parameters for the Langevin equation. This is in agreement with models of accumulation of evidence, where a single Langevin equation is used, that is parameters are not changed across trials in the simulation of behavioral data. For the LSA, *g*(*z*) and *h*(*z*) were fit to polynomials of order 1 and 0, respectively, and for the nonlinear model, they were fit to polynomials of order 3 and 2, respectively.

#### Simulations

Using the Langevin equation with parameters as explained in the previous paragraph, numerical simulations were performed for the chosen set of channels. *N*_*T*_ = 100 simulations were done for each value of the threshold crossing (*β*). The Euler-Maruyama method^24^ was used to numerically solve the Langevin equation, with constant discrete time step of Δt = 2^−8^ s. Each simulation started at the same initial condition *z*(0) = 0. A waiting time (WT) distribution was calculated for the ensemble of the simulations at a given *β*. WT was defined as the first time a given simulation had values within the interval |*z* − *β*| < 0.01.

Waiting time distributions were fit to the inverse Gaussian (or Wald) distribution^25^. The probability density function of this distribution is given by,

$$\rho (w;\mu ,\lambda )=\,{[\frac{\lambda }{2\pi {w}^{3}}]}^{1/2}exp\{\frac{-\lambda {(w-\mu )}^{2}}{2{\mu }^{2}w}\},$$

where *μ* is the mean of the distribution, *λ* is called the shape parameter and *w* are the values of WT. The inverse Gaussian distribution describes the distribution of arrival times of Brownian paths to a fixed threshold.

#### Optimization

The parameter *β* was varied in order to find the model with optimal WT distribution. The optimal WT distribution was chosen to be the closest to the WT distribution across all subjects and trials (that was fitted to an inverse Gaussian probability density function *ρ*(*w*; *M*, *L*), with *M* and *L*, the mean and shape parameters of the distribution, respectively). An optimization function was defined as the Euclidean distance between the WT distribution for all subjects and the WT distribution of the model for a given value of *β* in the (*μ*, *λ*) plane,

$$E(\beta )=\,\sqrt{{(\mu (\beta )-{\rm{{\rm M}}})}^{2}+{(\lambda (\beta )-L)}^{2}},$$

where *μ*(*β*) and λ(*β*) are the mean and shape parameter for a given threshold *β*. The value of *β* that best fit the WT distribution of all subjects was found by minimization of *E*,

$${\beta }_{0}={\rm{\arg }}\,{\rm{\min }}(E(\beta )).$$

The threshold (*β*) was varied until a minimum of *E*(*β*) was found, typically in the interval from *β* = 2 to *β* = 8. The optimal WT distribution is given by *ρ*_*opt*_ = *ρ*(*w*; *μ*(*β*_0_), *λ*(*β*_0_)).

Finally, the LSA and nonlinear models used to describe the complete dataset (Figs 8, 9) were the models that achieve the minimal value of *E* across subjects.

#### Distance between statistical distributions

The optimal WT distributions for the nonlinear and the LSA model were compared with the empirical WT distribution. To quantify this comparison, a distance between statistical distributions was used. We used a conventional metric, the scalar product between two real functions *f*(*x*) and *g*(*x*) in Hilbert space^26^, defined as

$$\langle f,g\rangle ={\int }_{-\infty }^{\infty }f(x)g(x)dx.$$

Using this metric, we calculated the distance between two statistical distributions *ρ*_1_(*w*) and *ρ*_2_ (*w*) as,

$$D({\rho }_{1},{\rho }_{2})=\sqrt{{\int }_{-\infty }^{\infty }{({\rho }_{1}(w)-{\rho }_{2}(w))}^{2}dw}.$$

All the algorithms were coded in MATLAB^©^.

## Supplementary information

Supplementary Information

## Data Availability

The datasets generated during and/or analysed during the current study are available from the corresponding author on reasonable request
