# Supplementary material for: An integration-to-bound model of decision-making that accounts for the spectral properties of neural data
Source: Sci Rep. 2019 Jun 10;9:8365. doi: 10.1038/s41598-019-44197-0 (PMC6557846; doi:10.1038/s41598-019-44197-0)

# An integration-to-bound model of decision-making that accounts for the spectral properties of neural data

Ramón Guevara Erra [1,2*], Marco Arbotto[3], Aaron Schurger [4, 5, 6, 7, 8]

[1] Laboratoire Psychologie de la Perception, UMR 8242, Université Paris Descartes, Paris, France.
[2] Laboratoire Psychologie de la Perception, UMR 8242, CNRS, Paris, France.
[3] Master of Biomedical Engineering, Paris Descartes University, Paris, France.
[4] INSERM, Cognitive Neuroimaging Unit, Gif sur Yvette 91191, France.
[5] Commissariat à l'Energie Atomique, Direction des Sciences du Vivant, I2BM, NeuroSpin center, Gif sur Yvette 91191, France.
[6] Laboratory of Cognitive Neuroscience, Brain Mind Institute, Department of Life Sciences, Ecole Polytechnique Fédérale de Lausanne, 1015, Lausanne, Switzerland.
[7] Defitech Chair in Non-Invasive Brain-Machine Interface, Center for Neuroprosthetics, School of Engineering, Ecole Polytechnique Fédérale de Lausanne, Lausanne, Switzerland
[8] Center for Neuroprosthetics, Ecole Polytechnique Fédérale de Lausanne, 1015, Lausanne, Switzerland.

* Corresponding author:
E-mail: guevarra.erra@gmail.com

## Supplementary material figure captions

**Fig S1. Deterministic part of the Langevin equation (Case 2).** Single trials (Case 2) reconstructed Langevin functions *g(x)* for all subjects, at all (magnetometer) channels, and corresponding best fits (hyperbolas). Each panel is a different subject. The Langevin functions are centered for the purpose of visualization (by shifting each Langevin function in $x$ by an amount $< x >$, where $< x >$ is the mean in $x$ of the data ensemble). Left: data (in blue), for all channels and all trials. Right: best fit (red) $g(x) = ax^3 + bx^2 + cx + d$, for each possible trial and channel.

**Fig S2. Stochastic part of the Langevin equation (Case 2).** Single trials (Case 2) reconstructed Langevin functions *h(x)* for all subjects, at all (magnetometer) channels, and corresponding best fits (parabolas). Each panel is a different subject. The Langevin functions are centered for the purpose of visualization (by shifting each Langevin function in $x$ by an amount $< x >$, where $< x >$ is the mean in $x$ of the data ensemble). Left: data (in blue), for all channels and all trials. Right: best fit (red) $h(x) = Ax^2 + Bx + C$, for each possible trial and channel.

Fig S1 Panel 1.

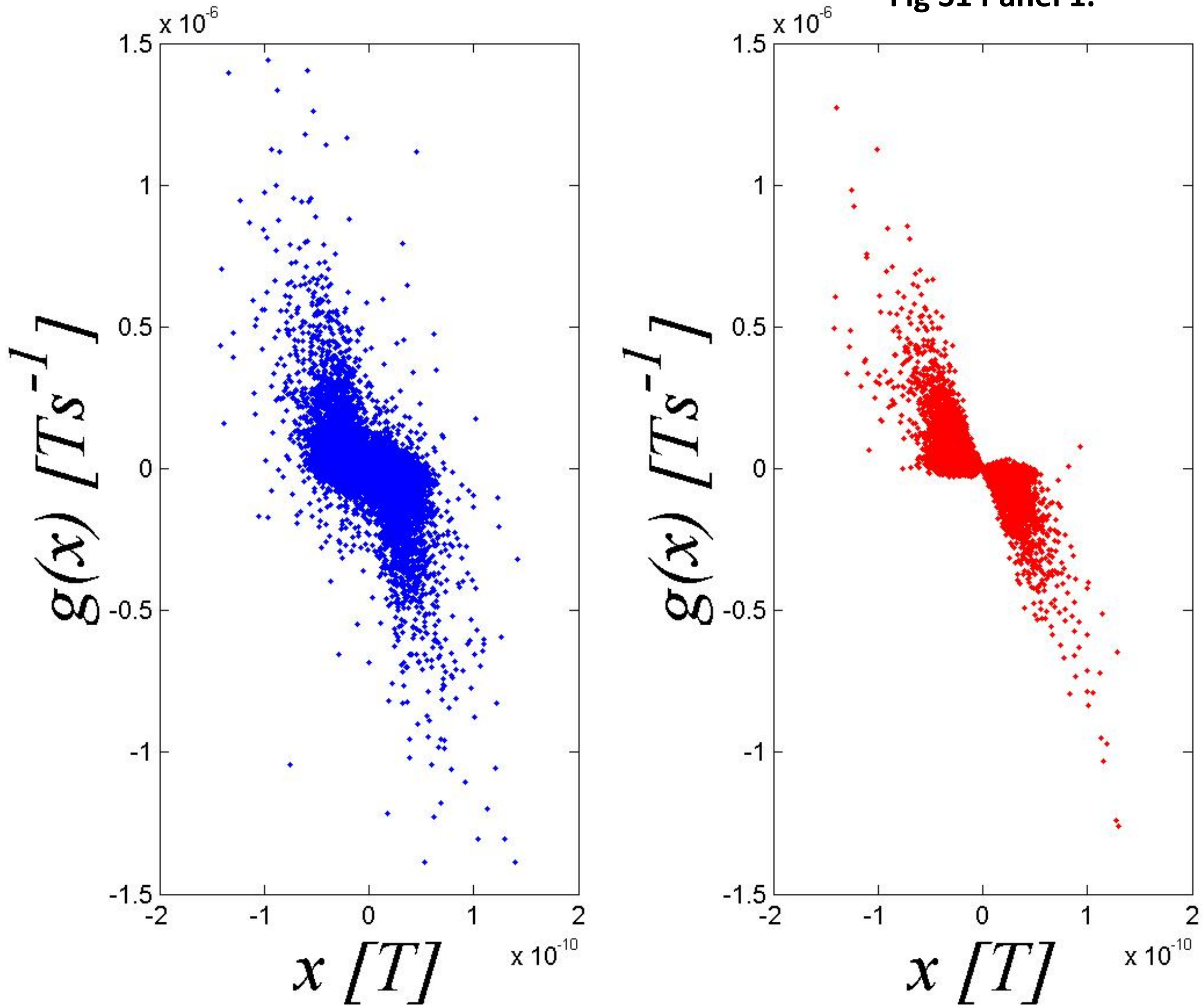

**Fig S1 Panel 2.**

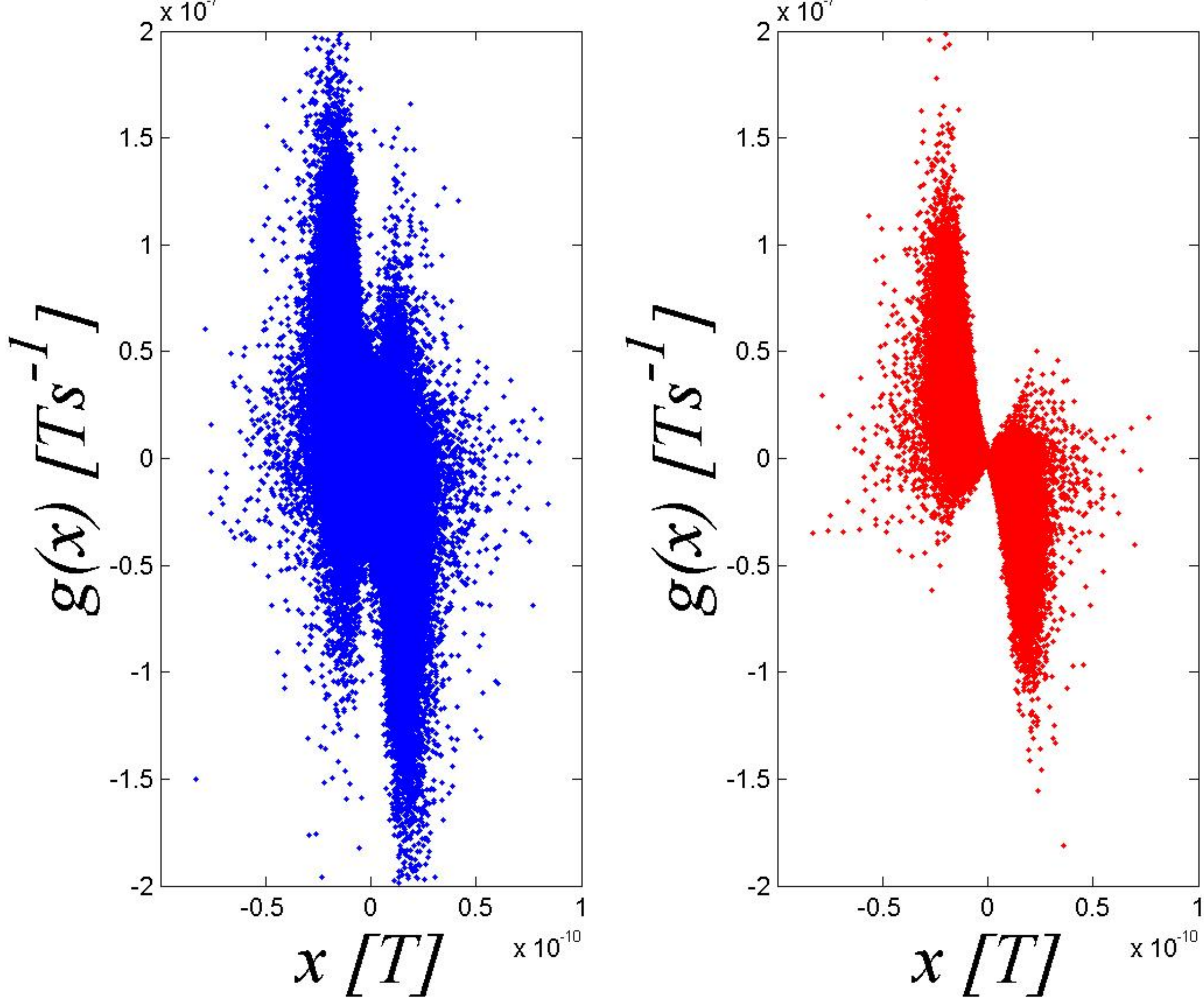

Fig S1 Panel 3.

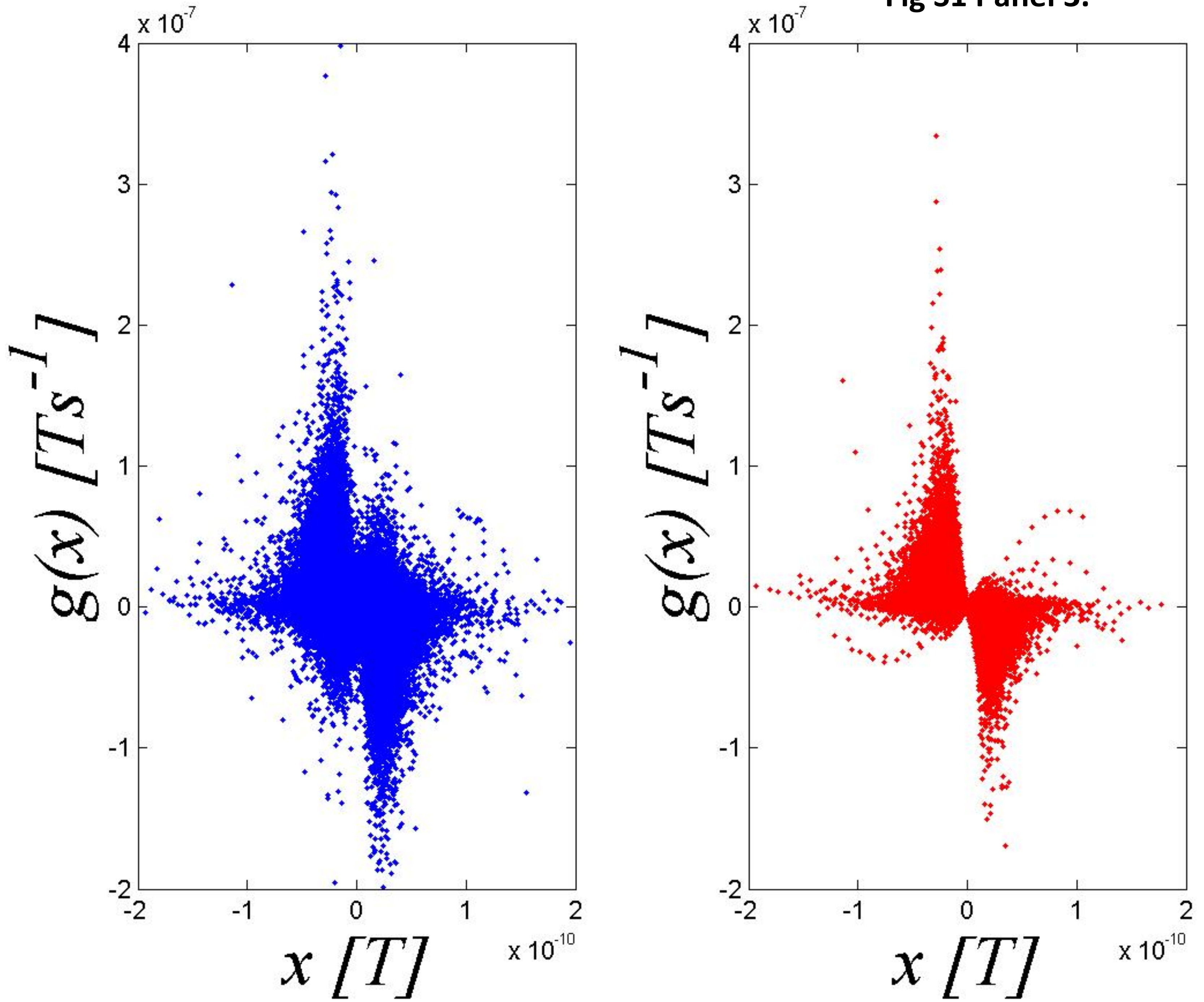

**Fig S1 Panel 4.**

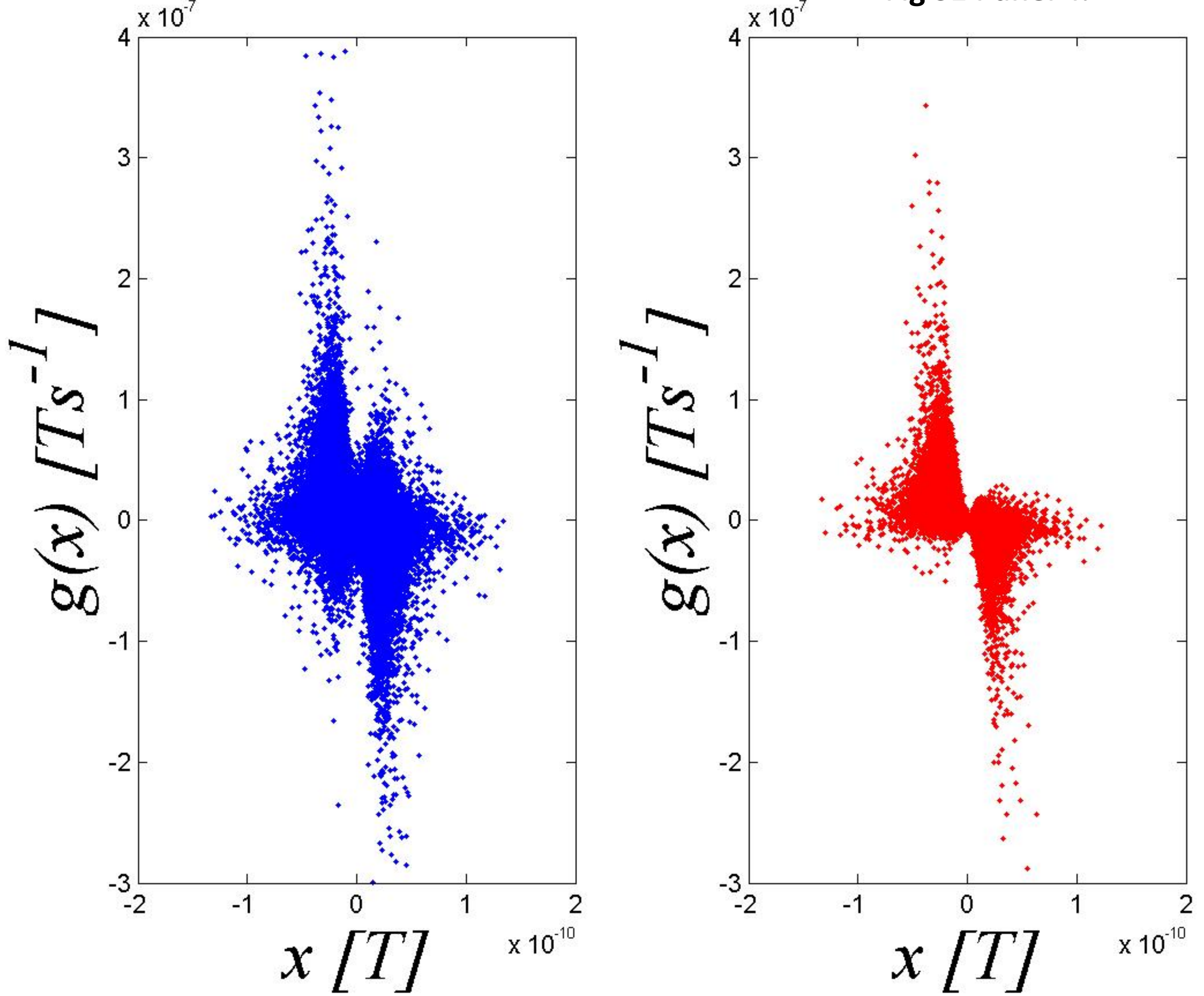

**Fig S1 Panel 5.**

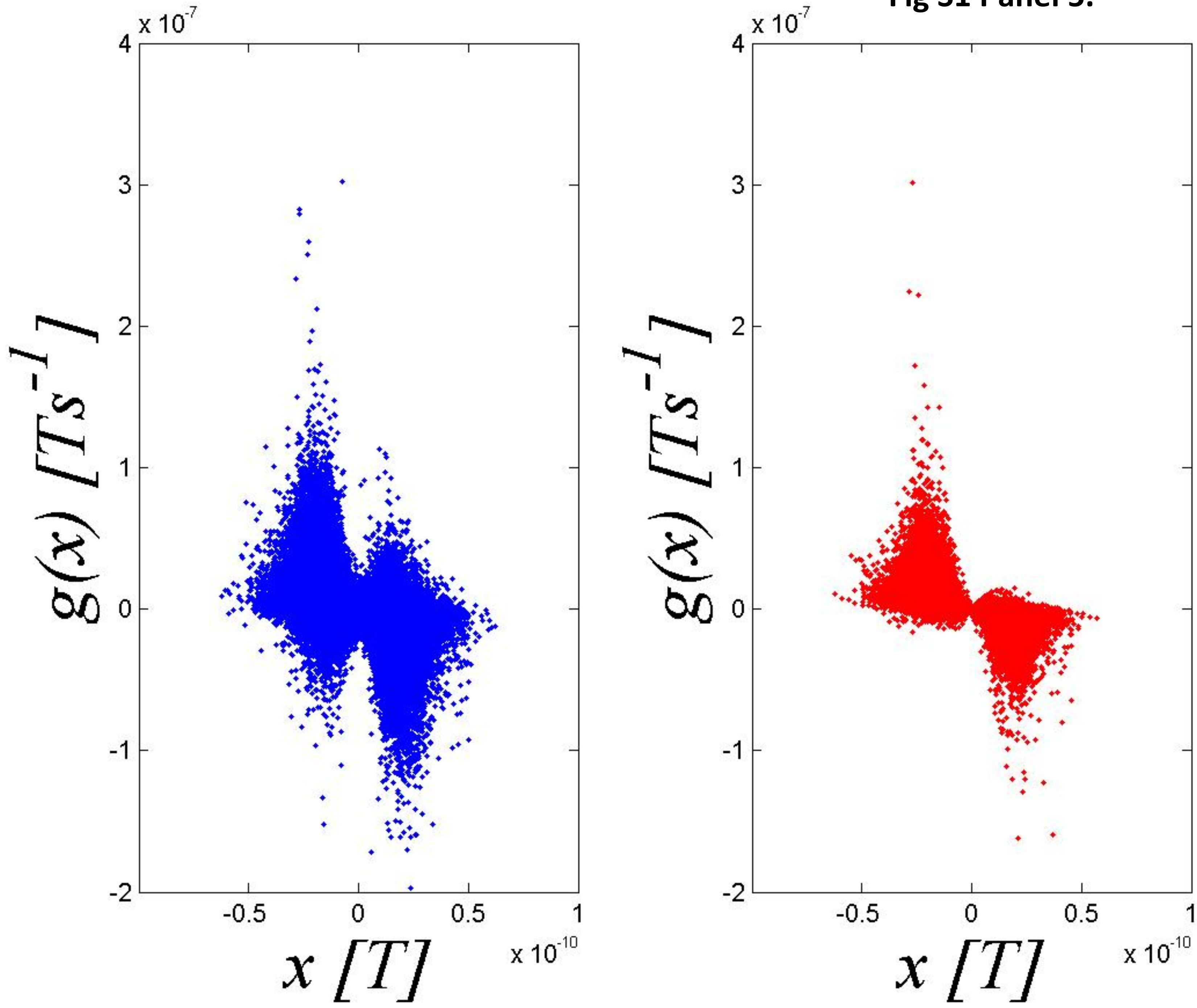

**Fig S1 Panel 6.**

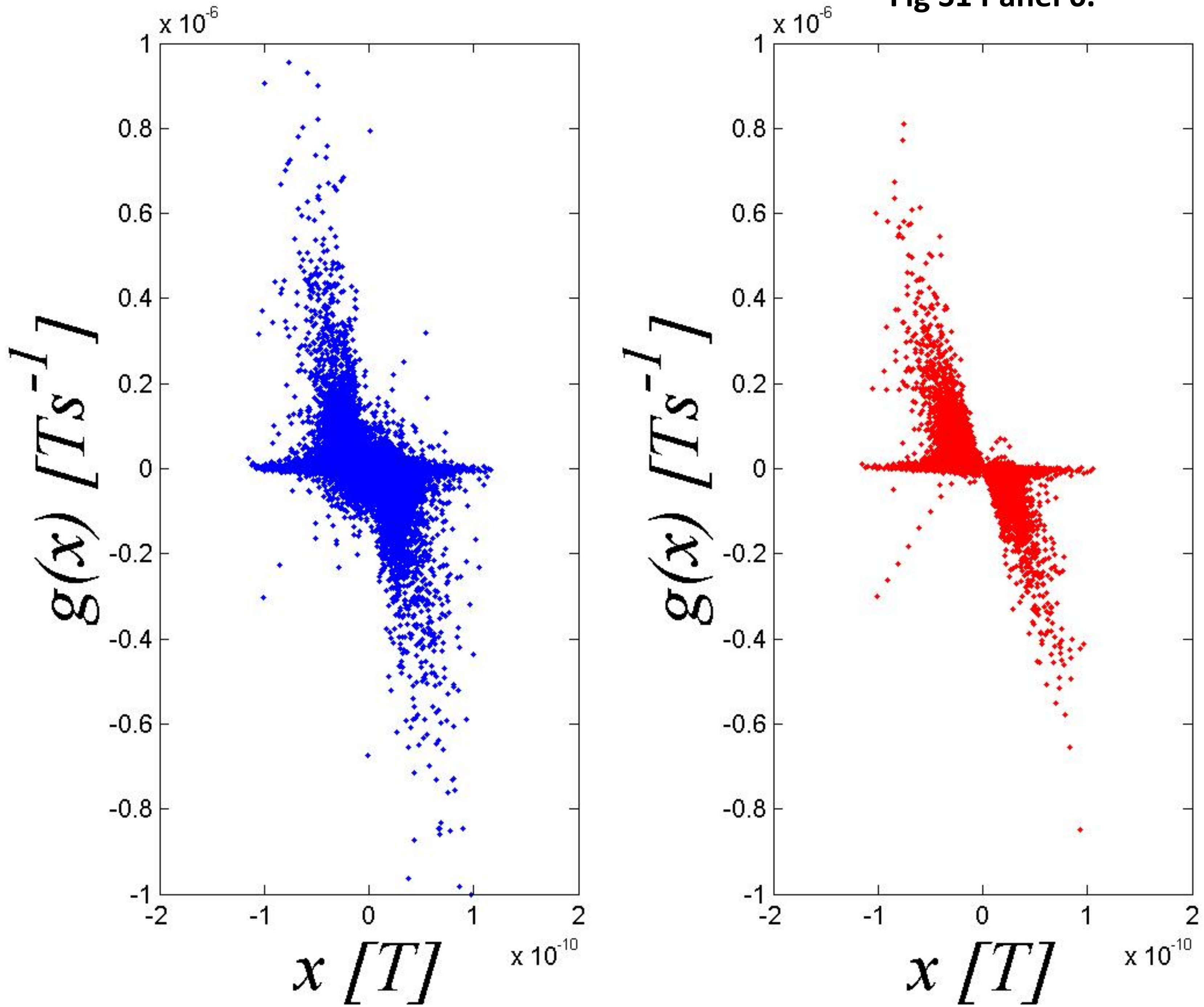

**Fig S1 Panel 7.**

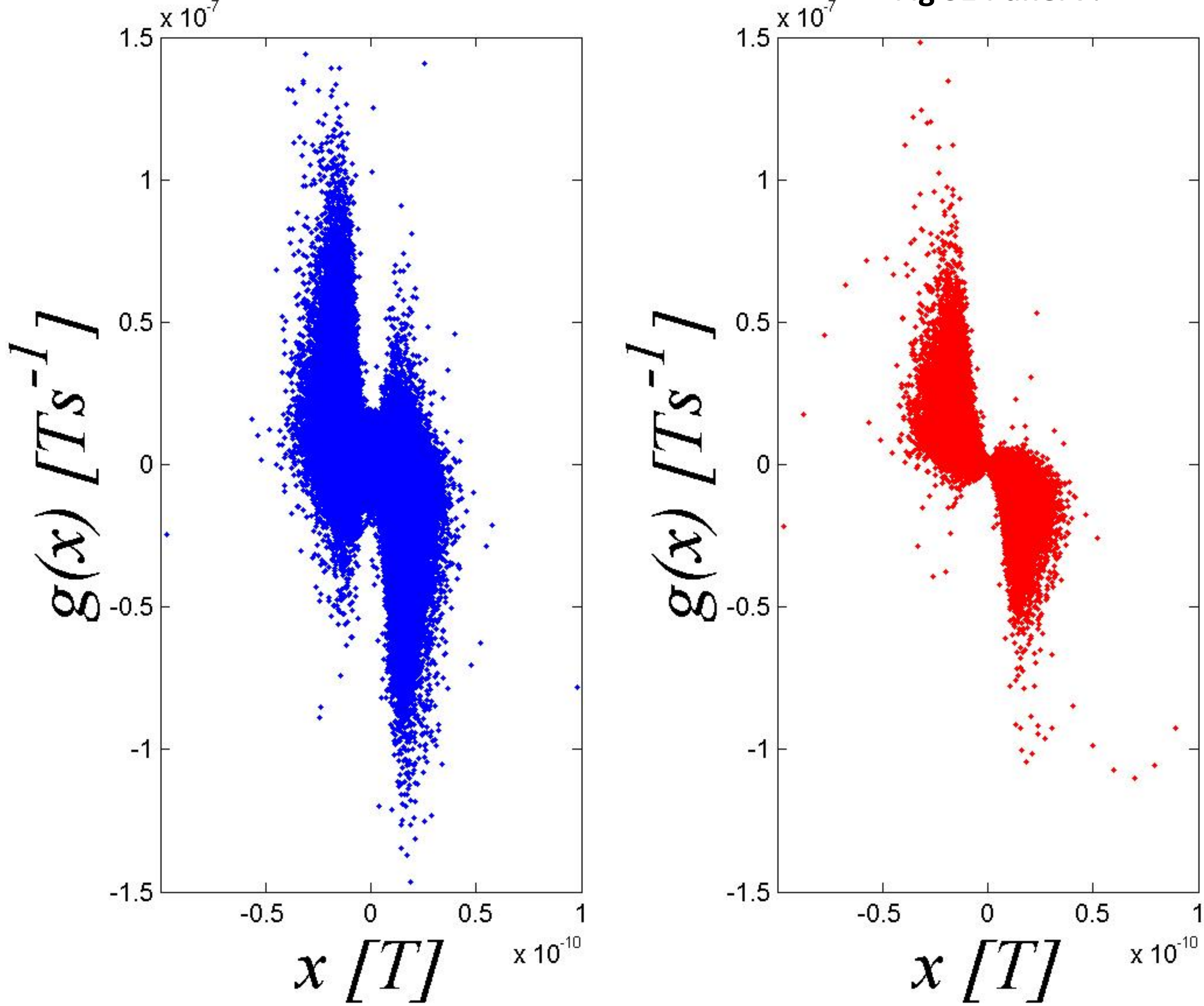

**Fig S1 Panel 8.**

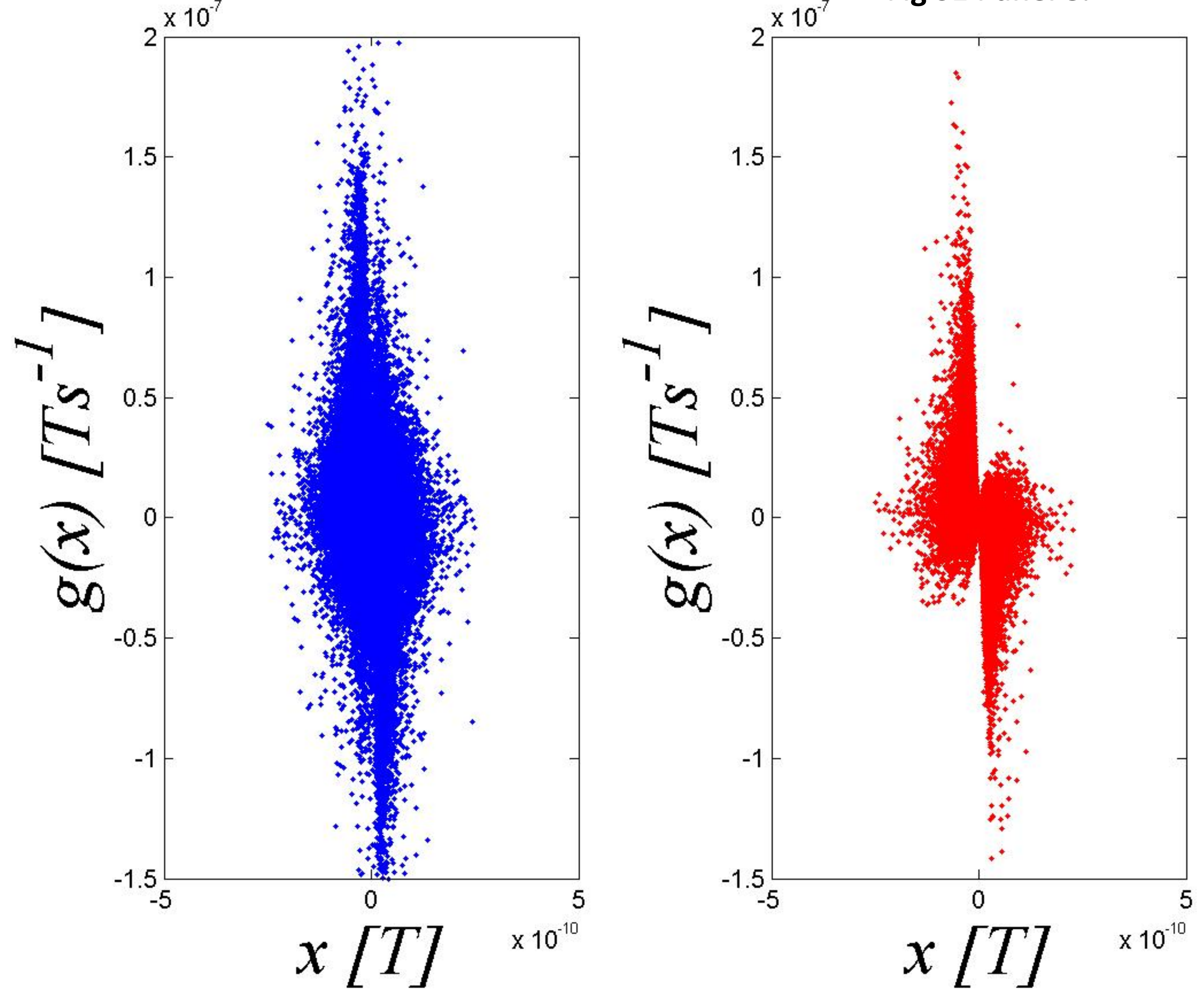

**Fig S1 Panel 9.**

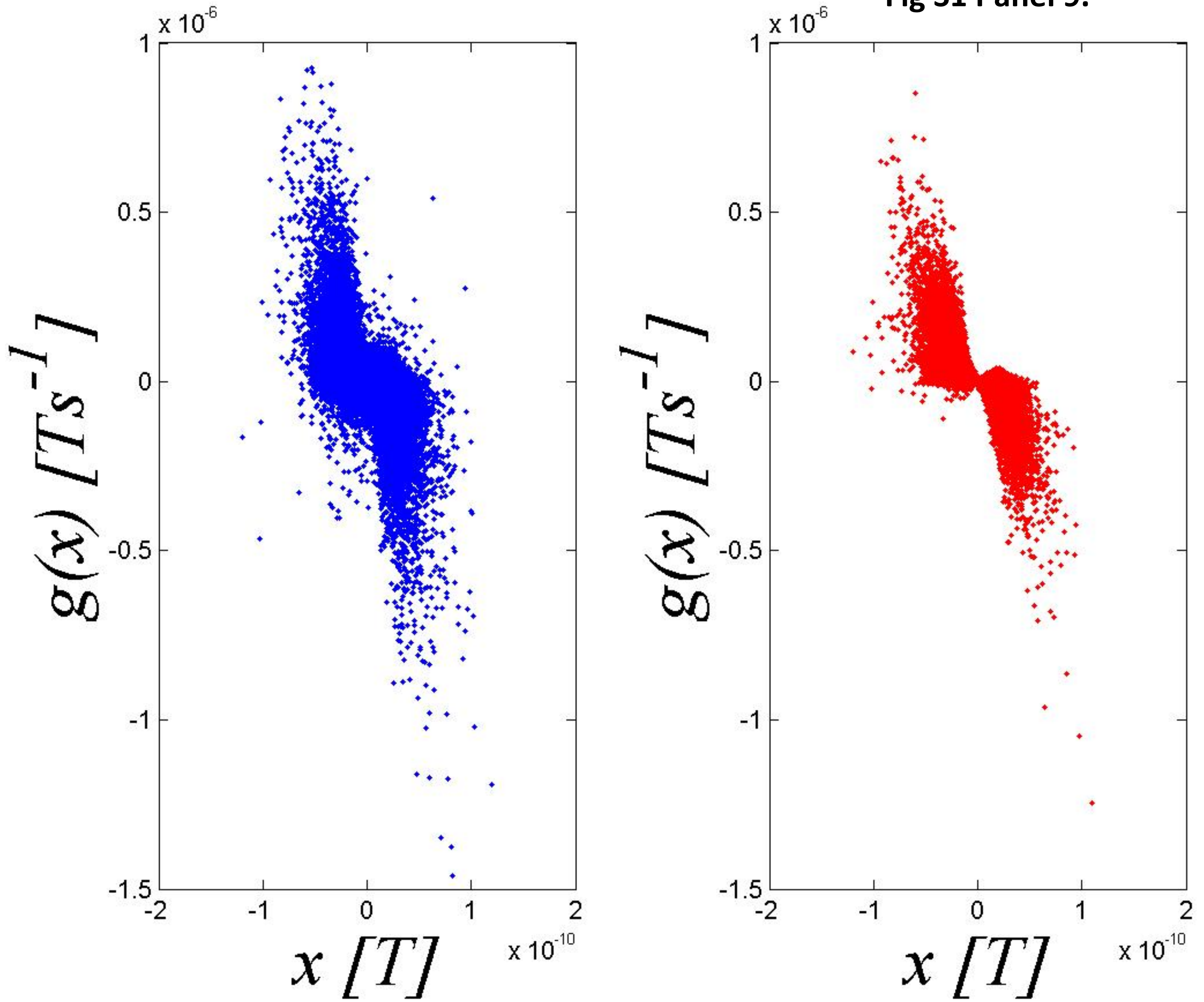

**Fig S1 Panel 10.**

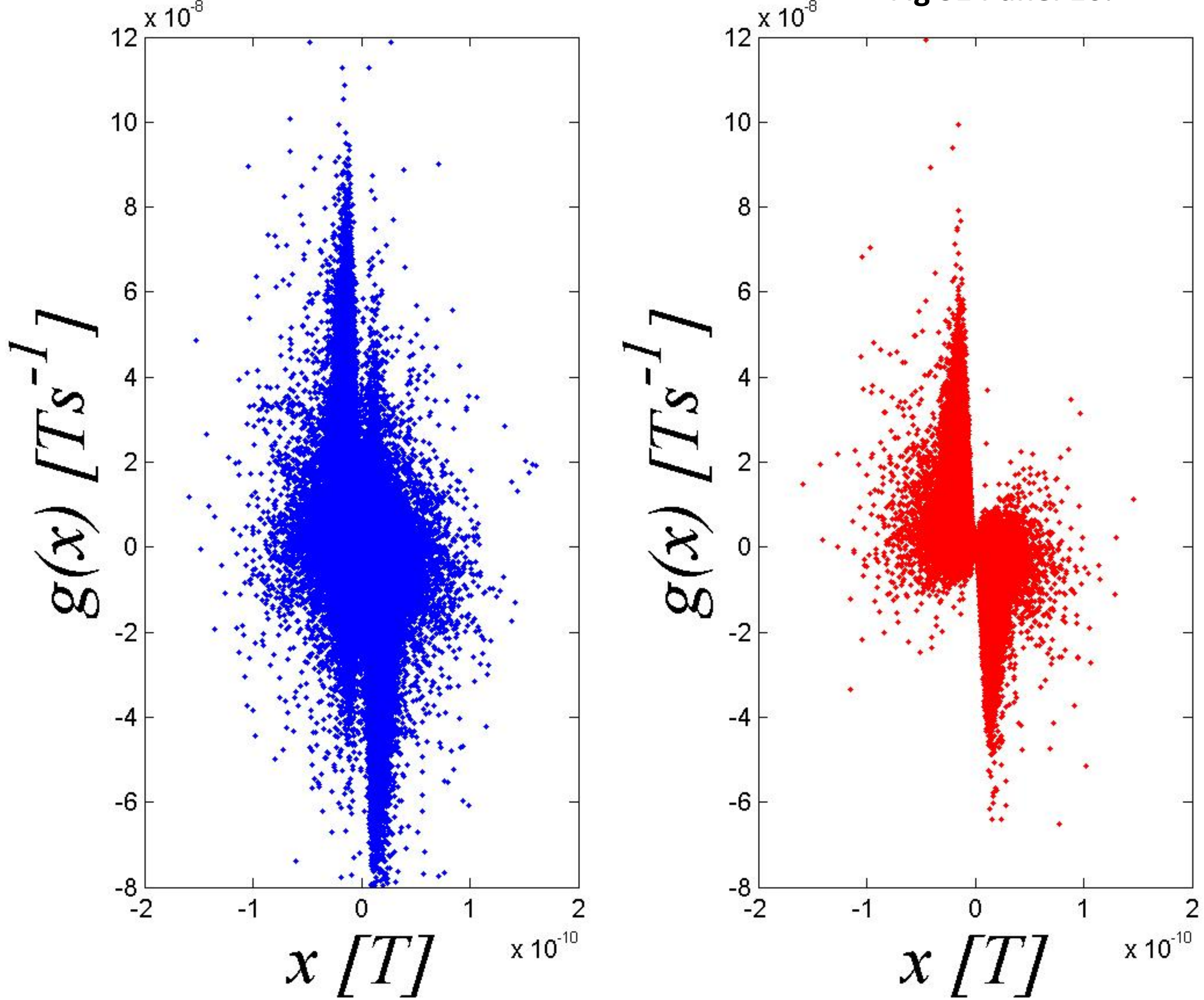

**Fig S1 Panel 11.**

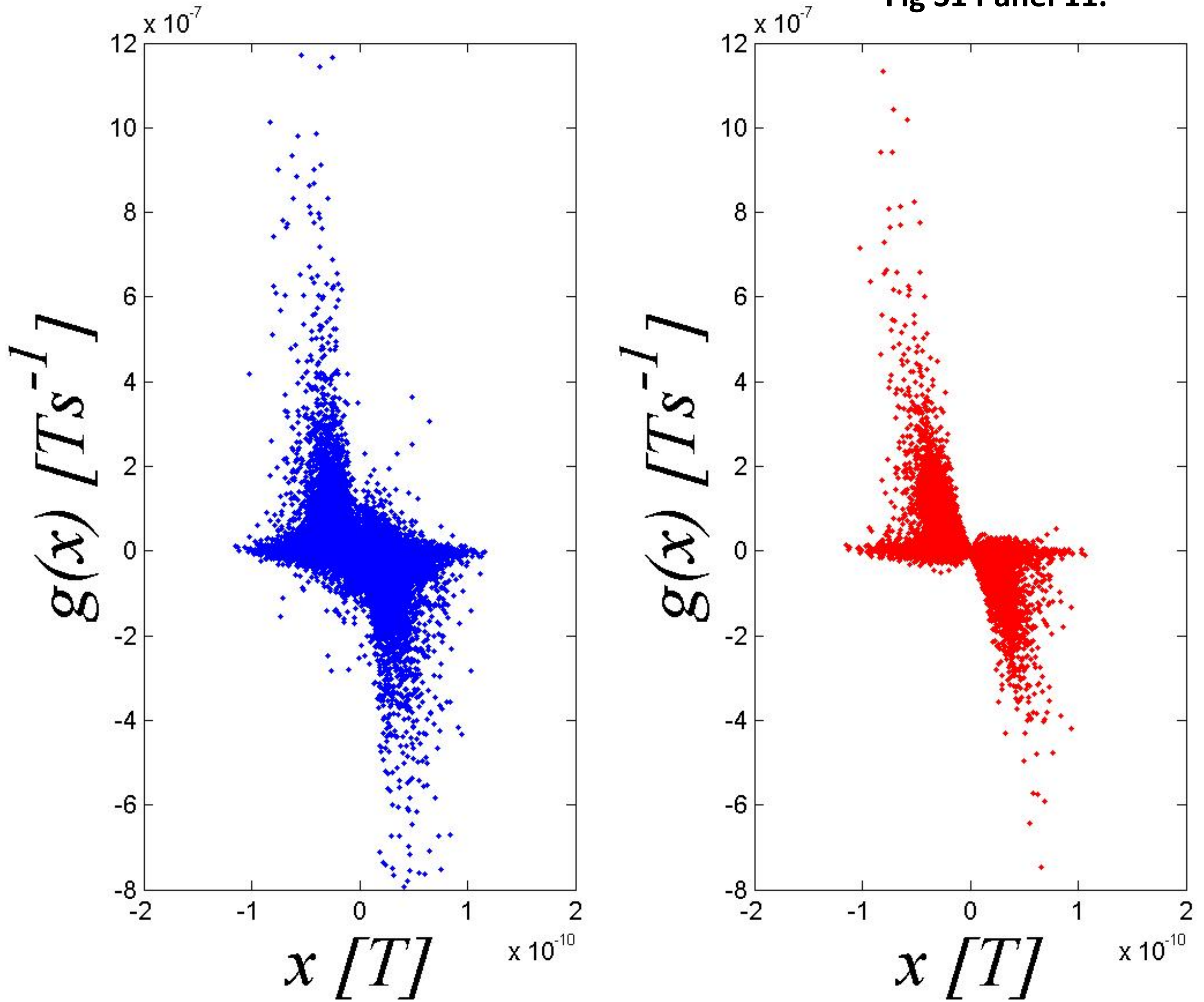

**Fig S1 Panel 12.**

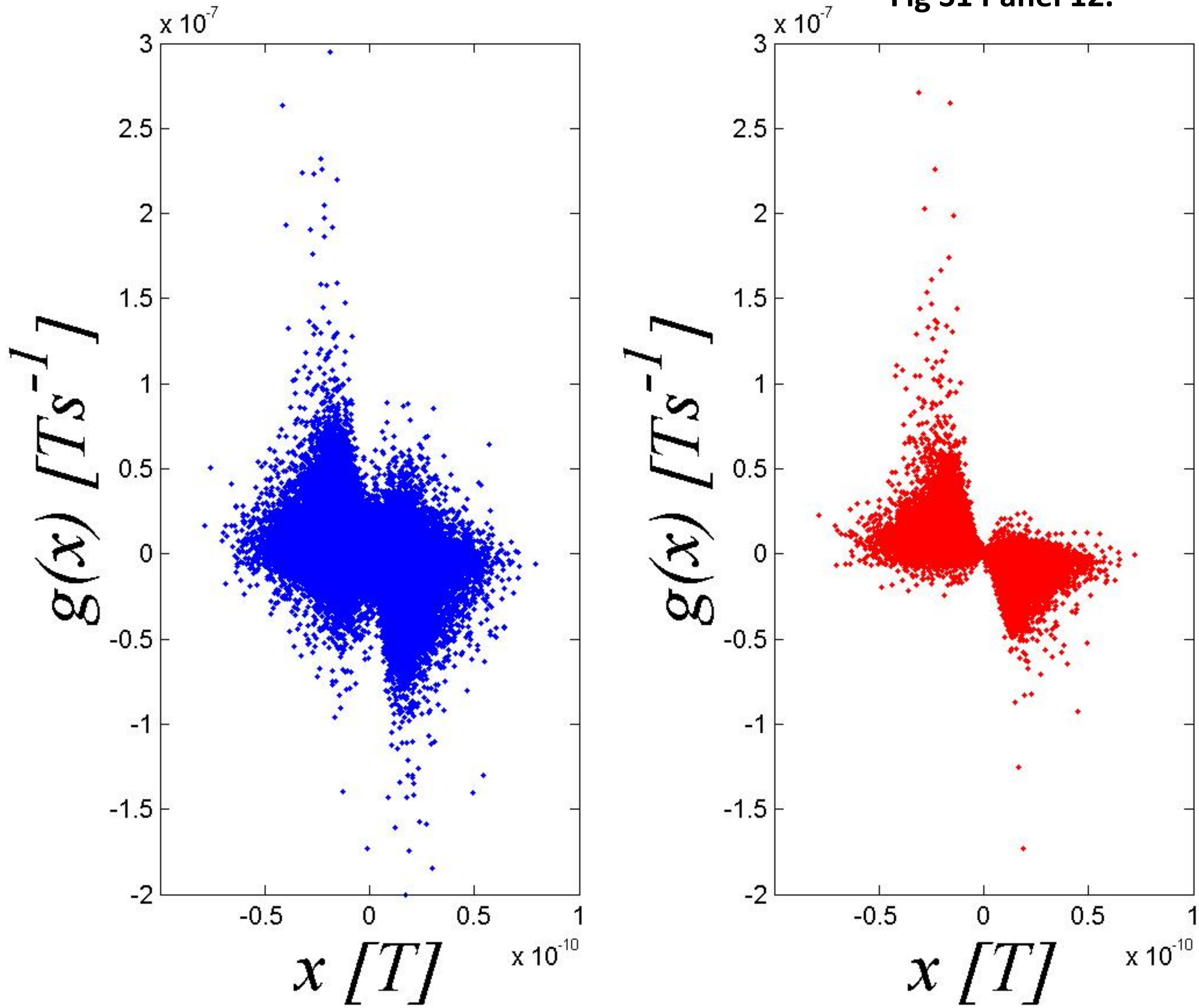

**Fig S1 Panel 13.**

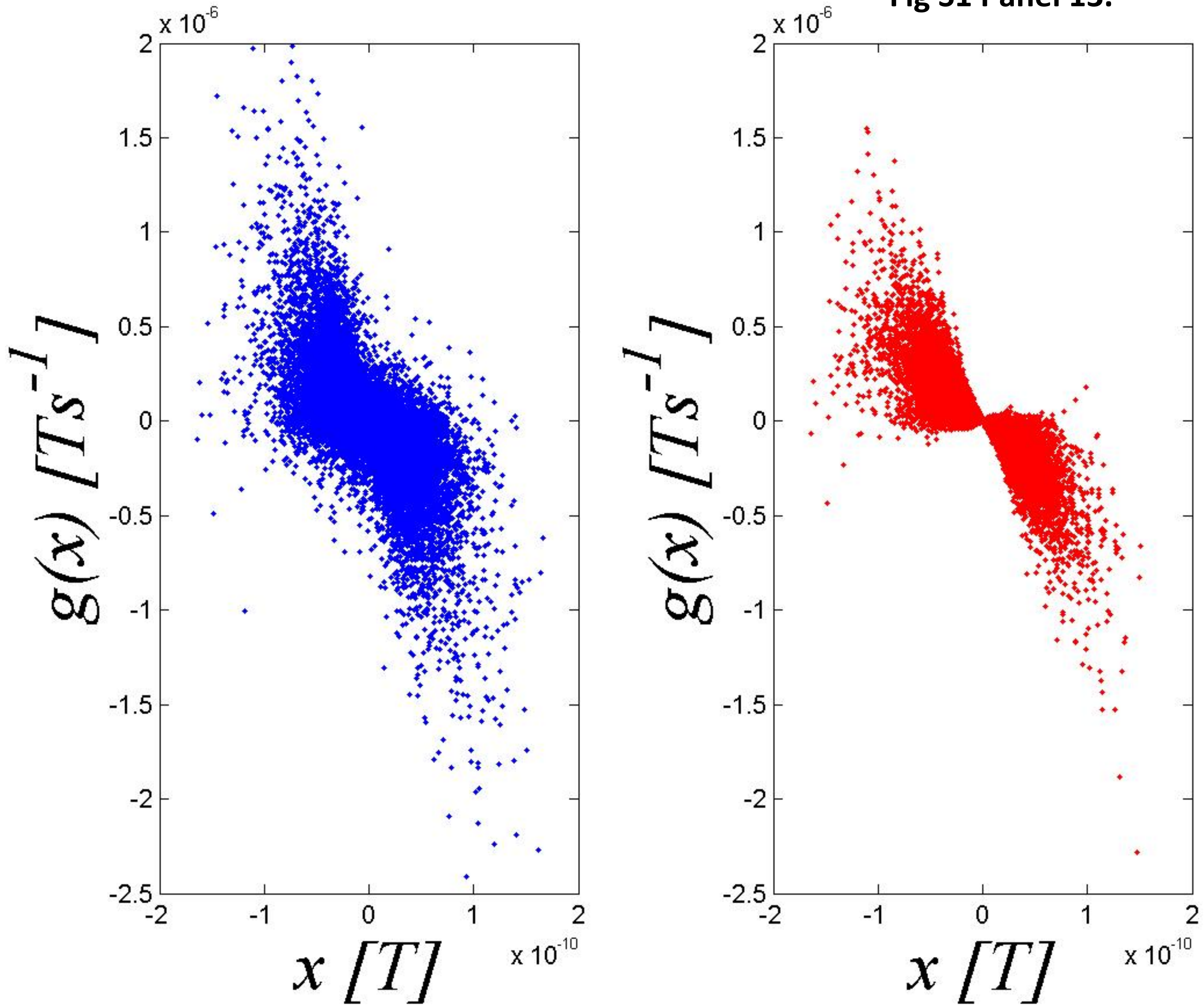

**Fig S1 Panel 14.**

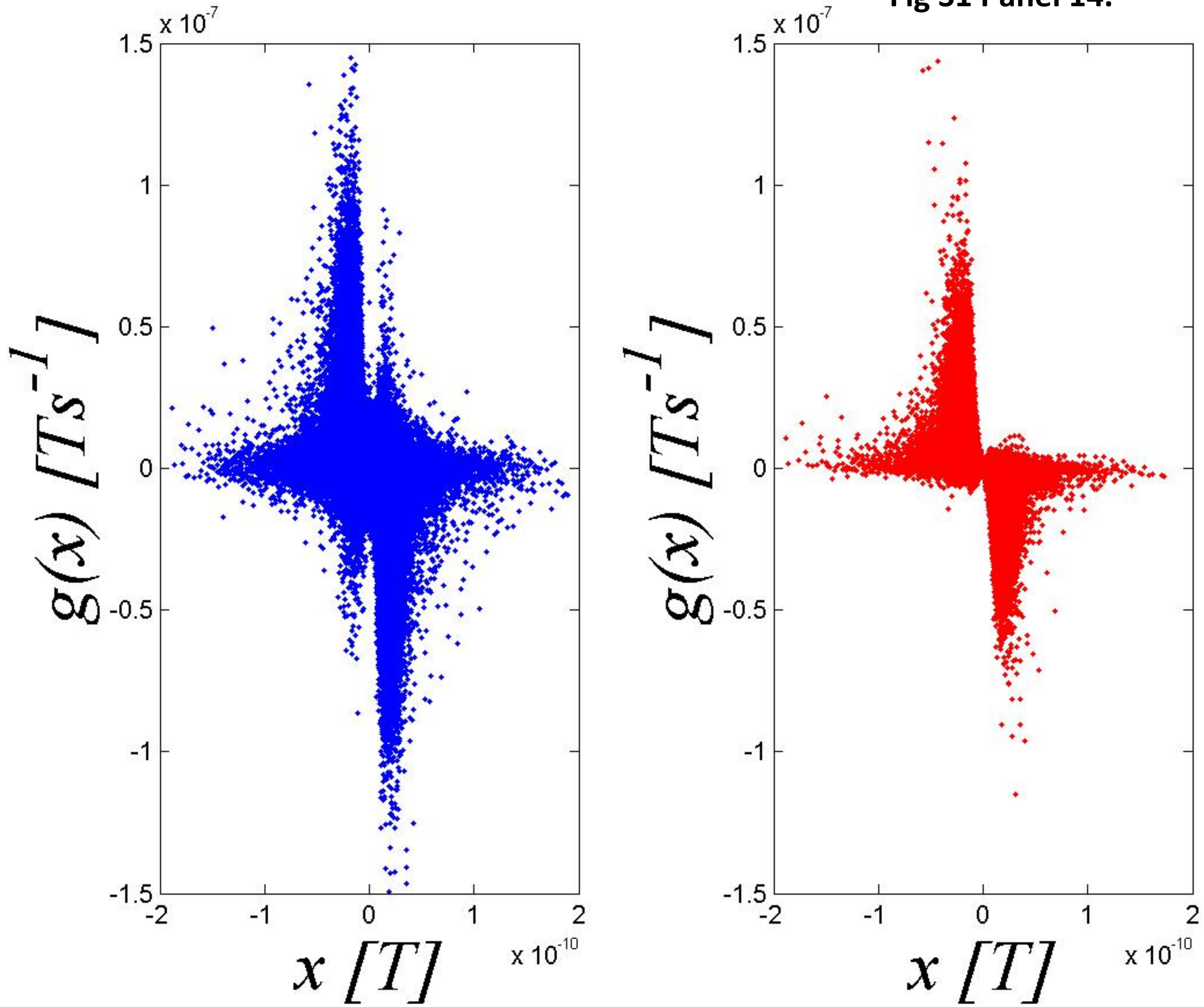

**Fig S2 Panel 1.**

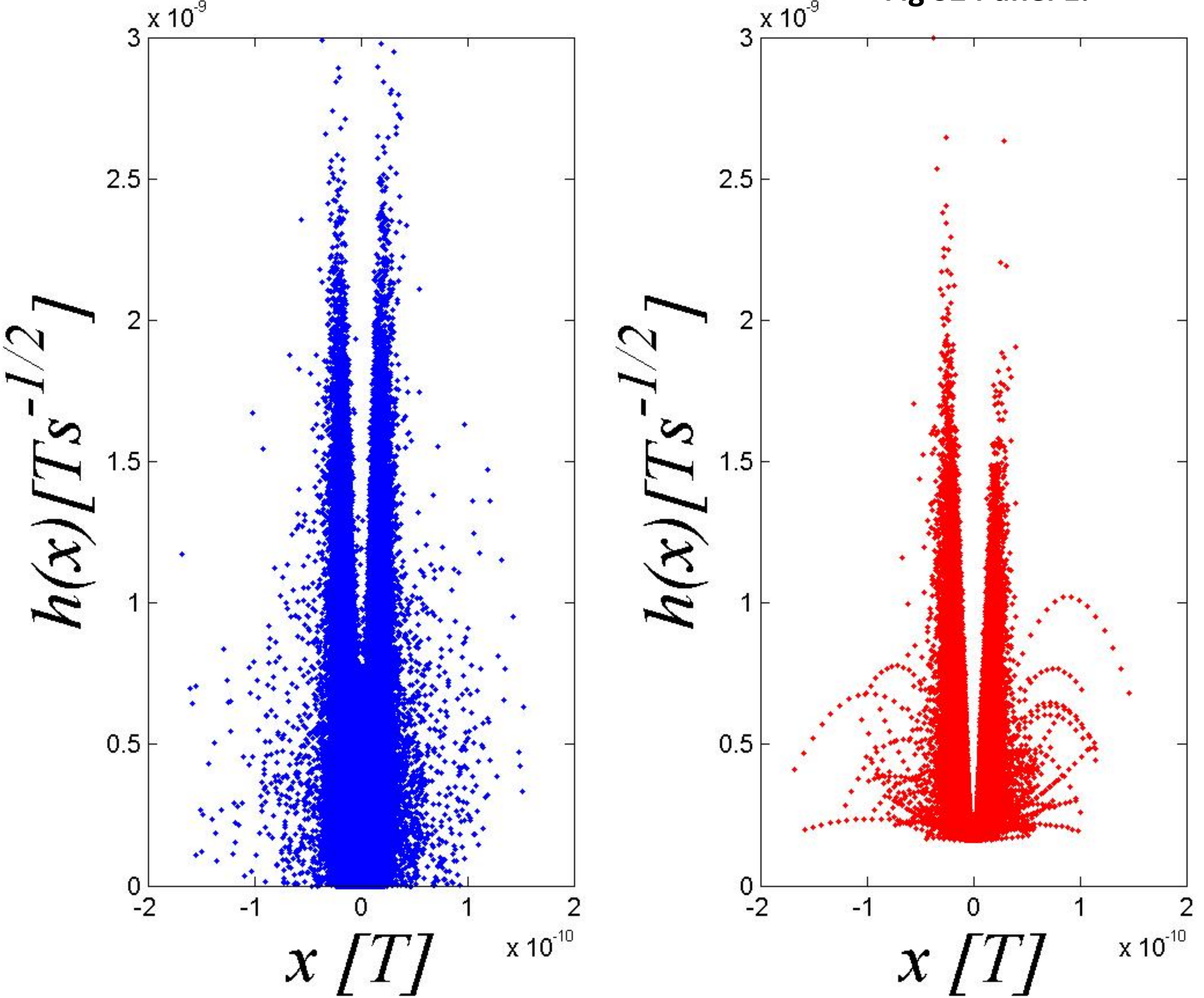

**Fig S2 Panel 2.**

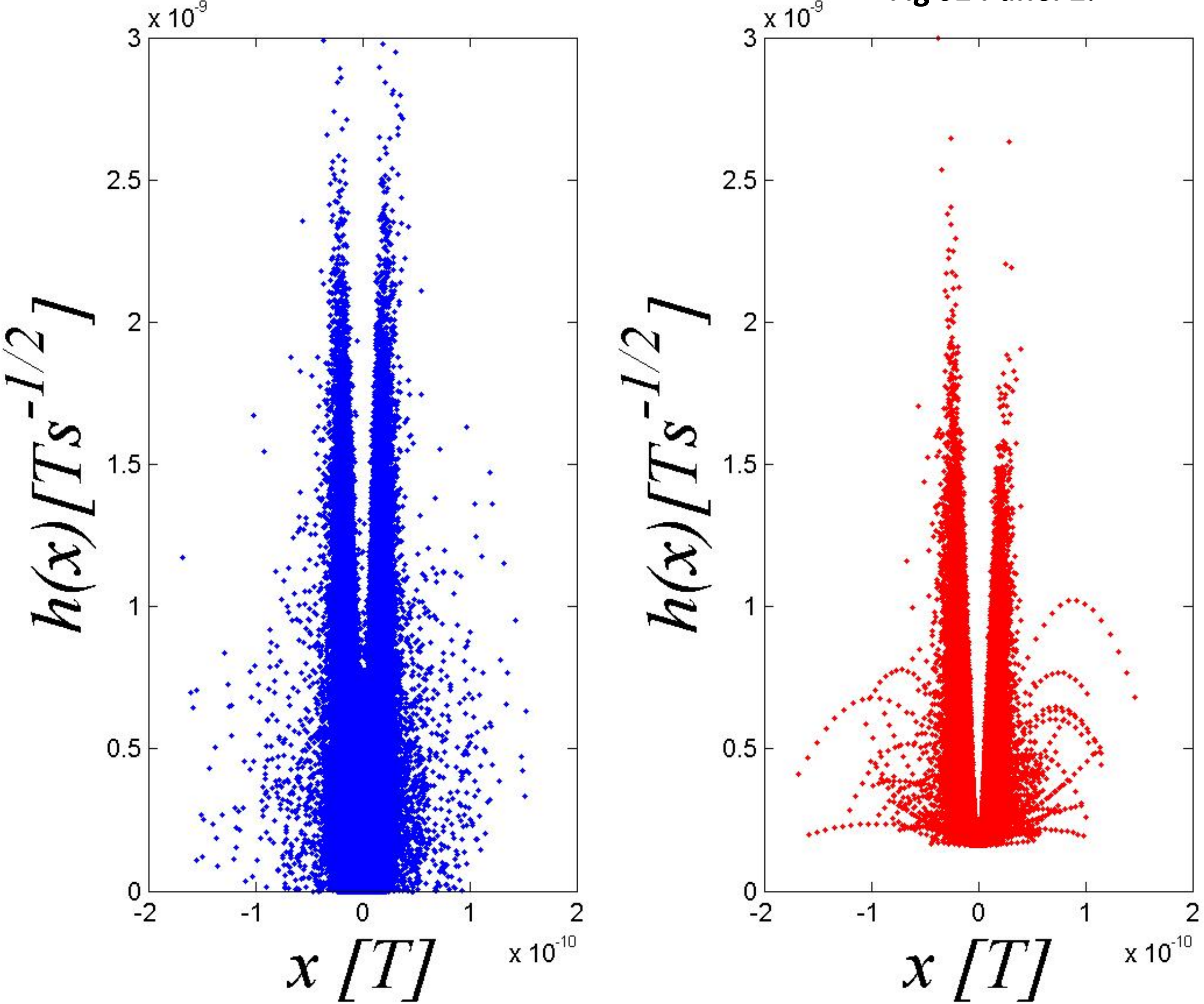

**Fig S2 Panel 3.**

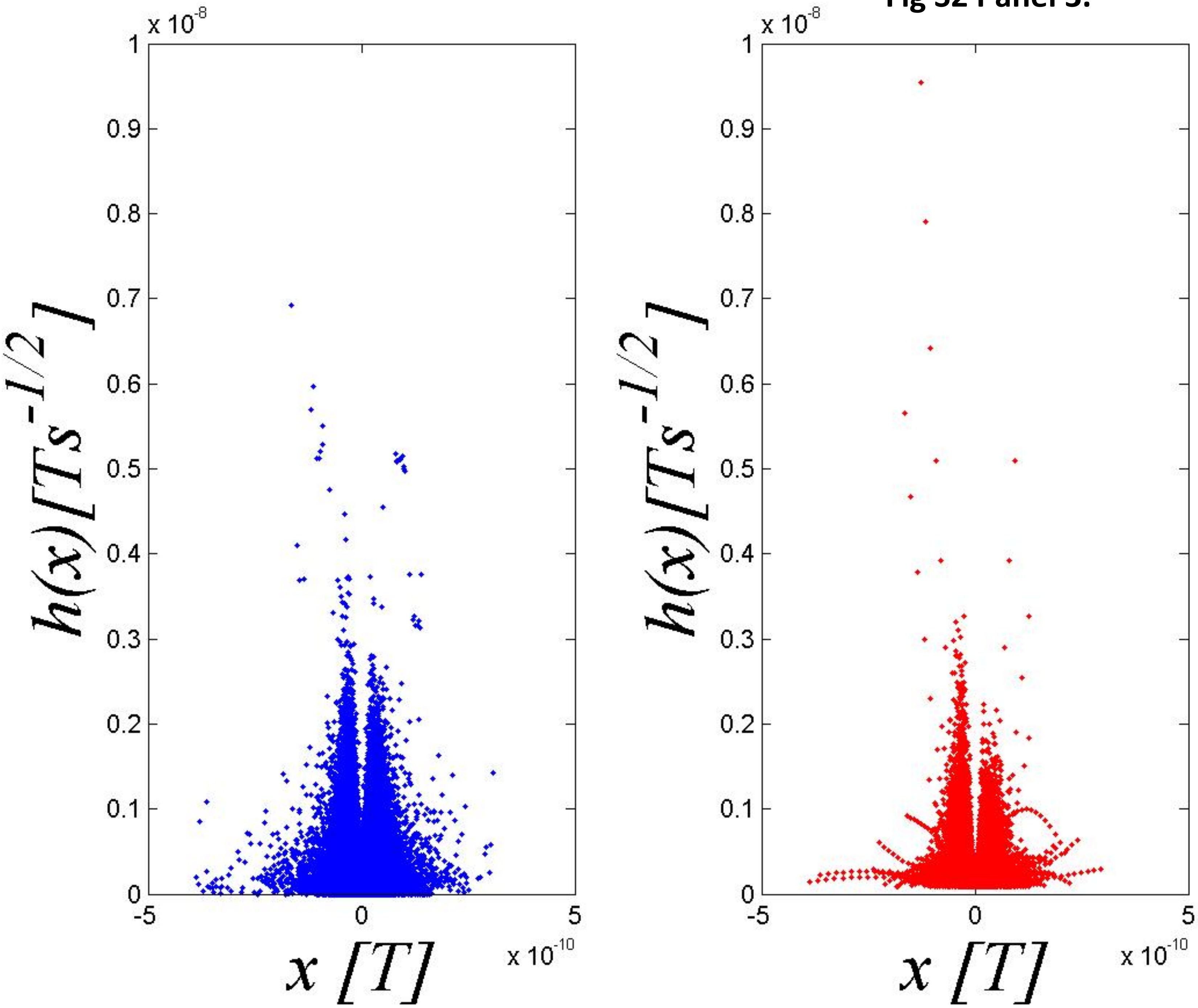

**Fig S2 Panel 4.**

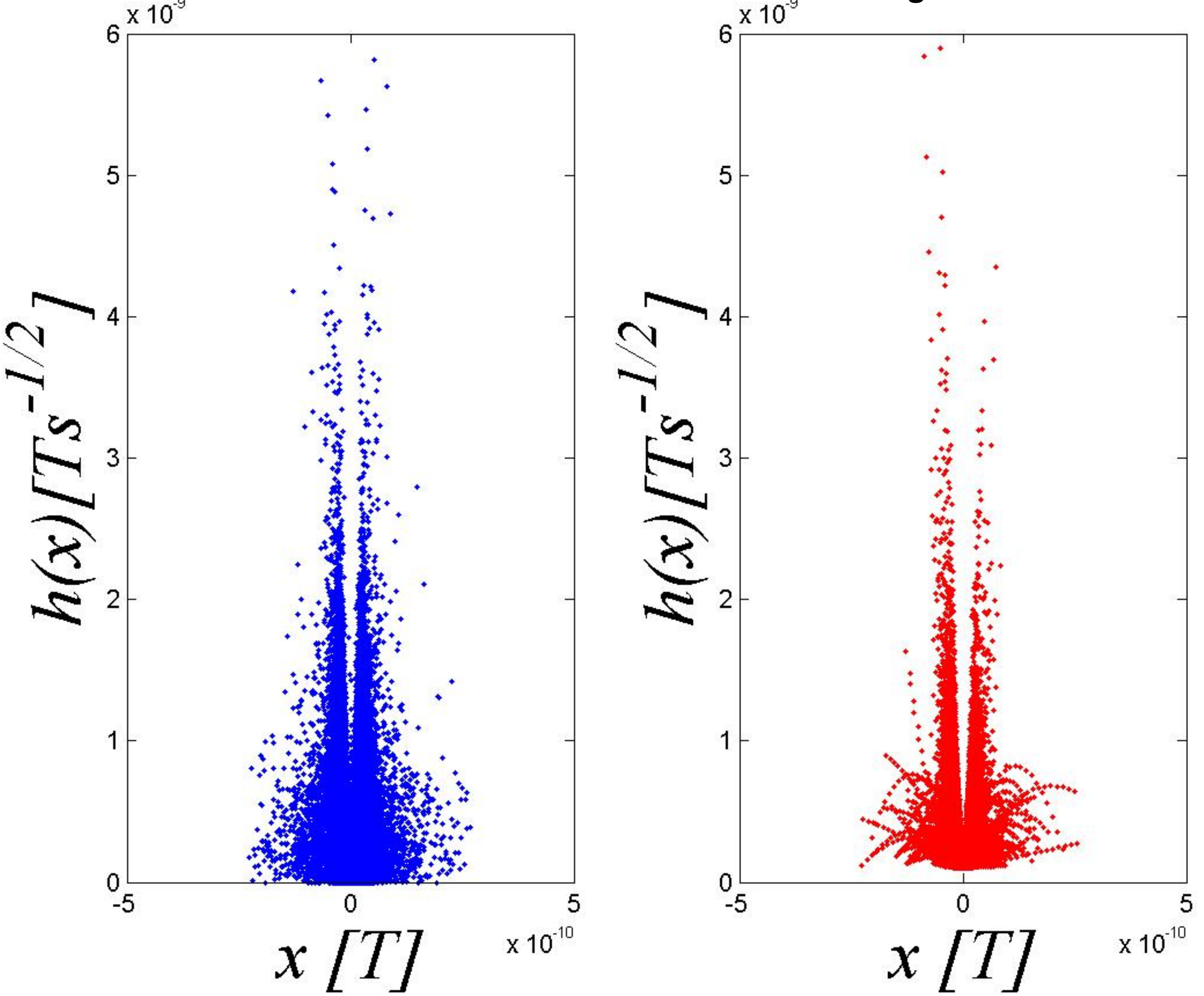

**Fig S2 Panel 5.**

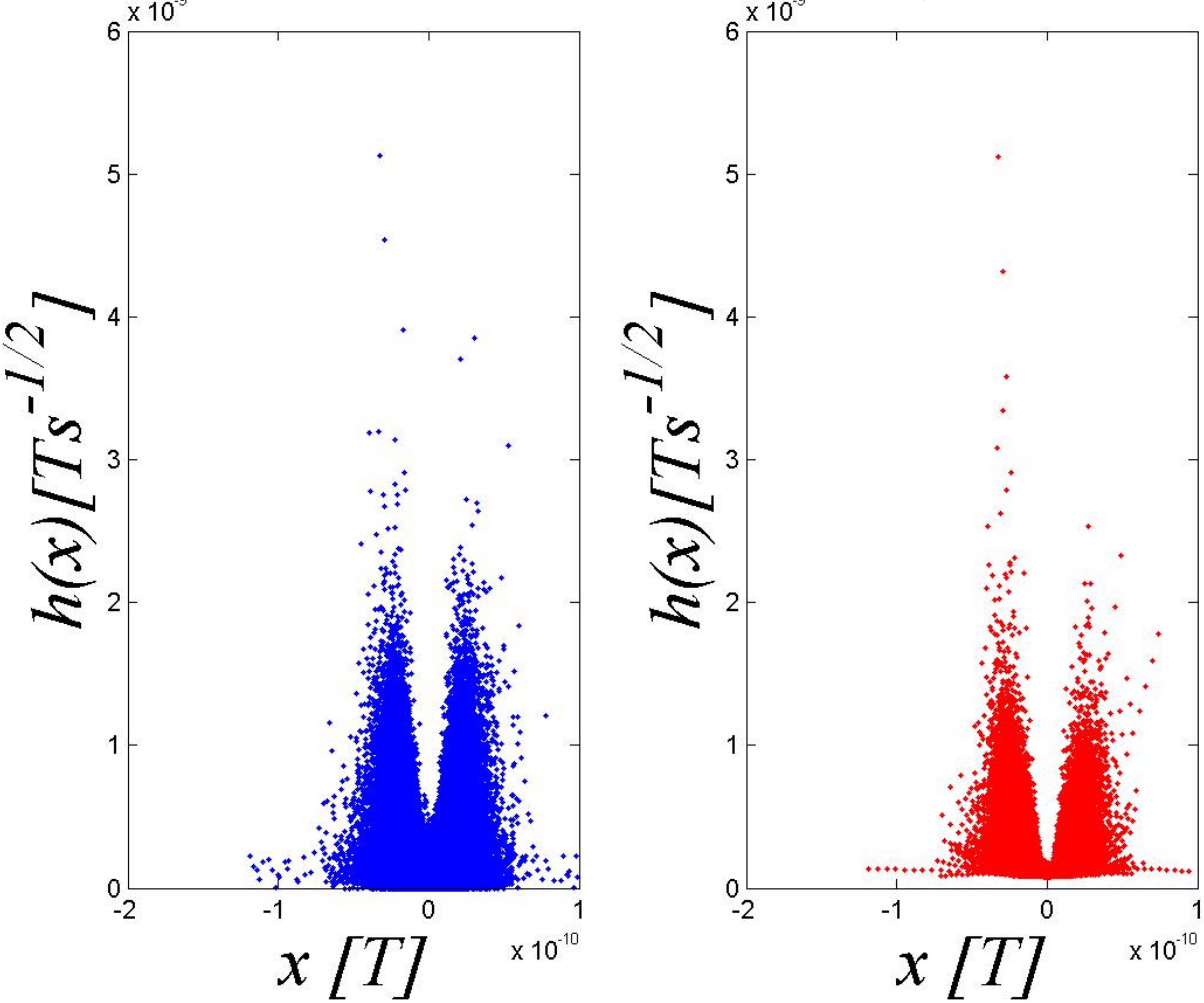

**Fig S2 Panel 6.**

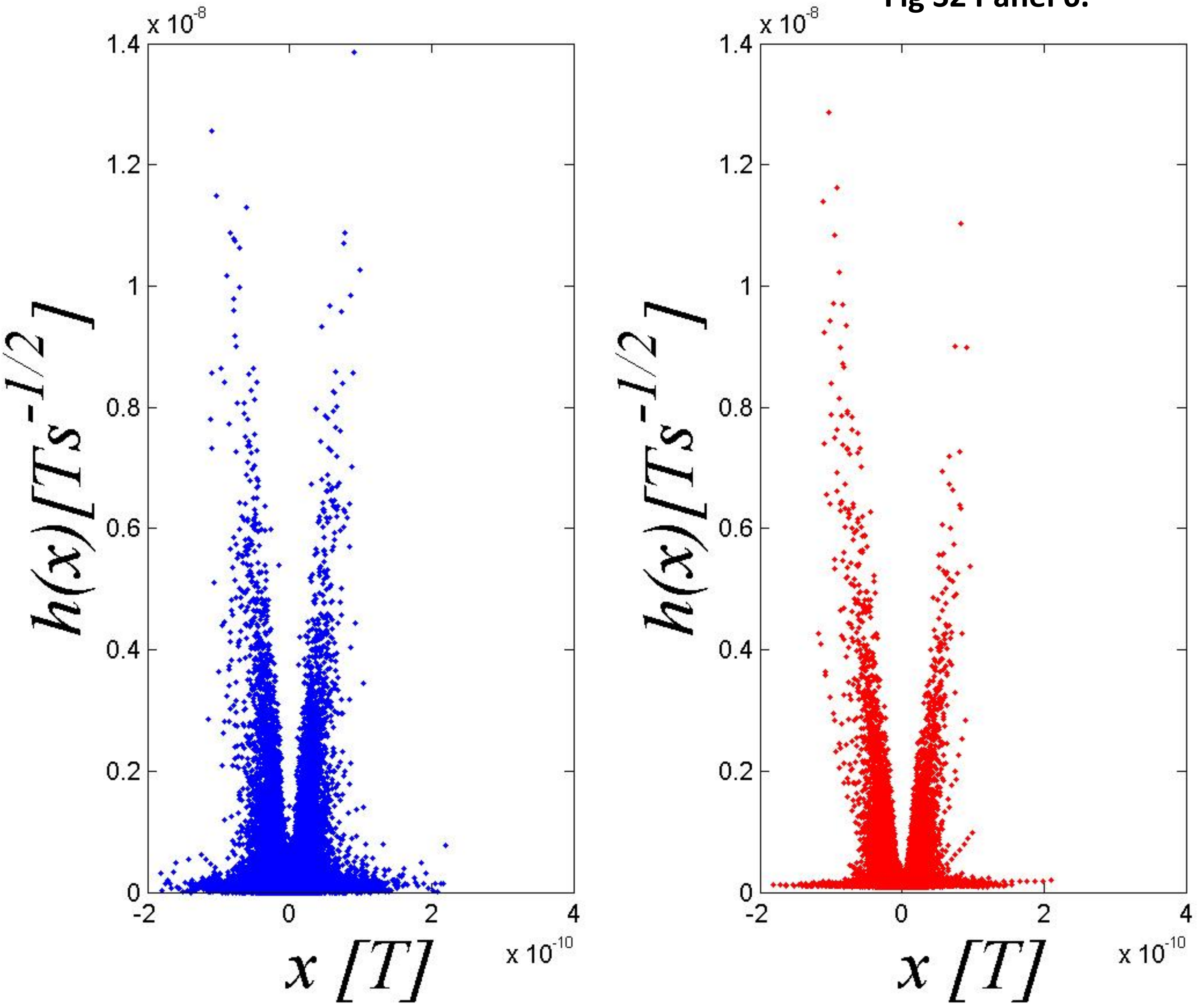

**Fig S2 Panel 7.**

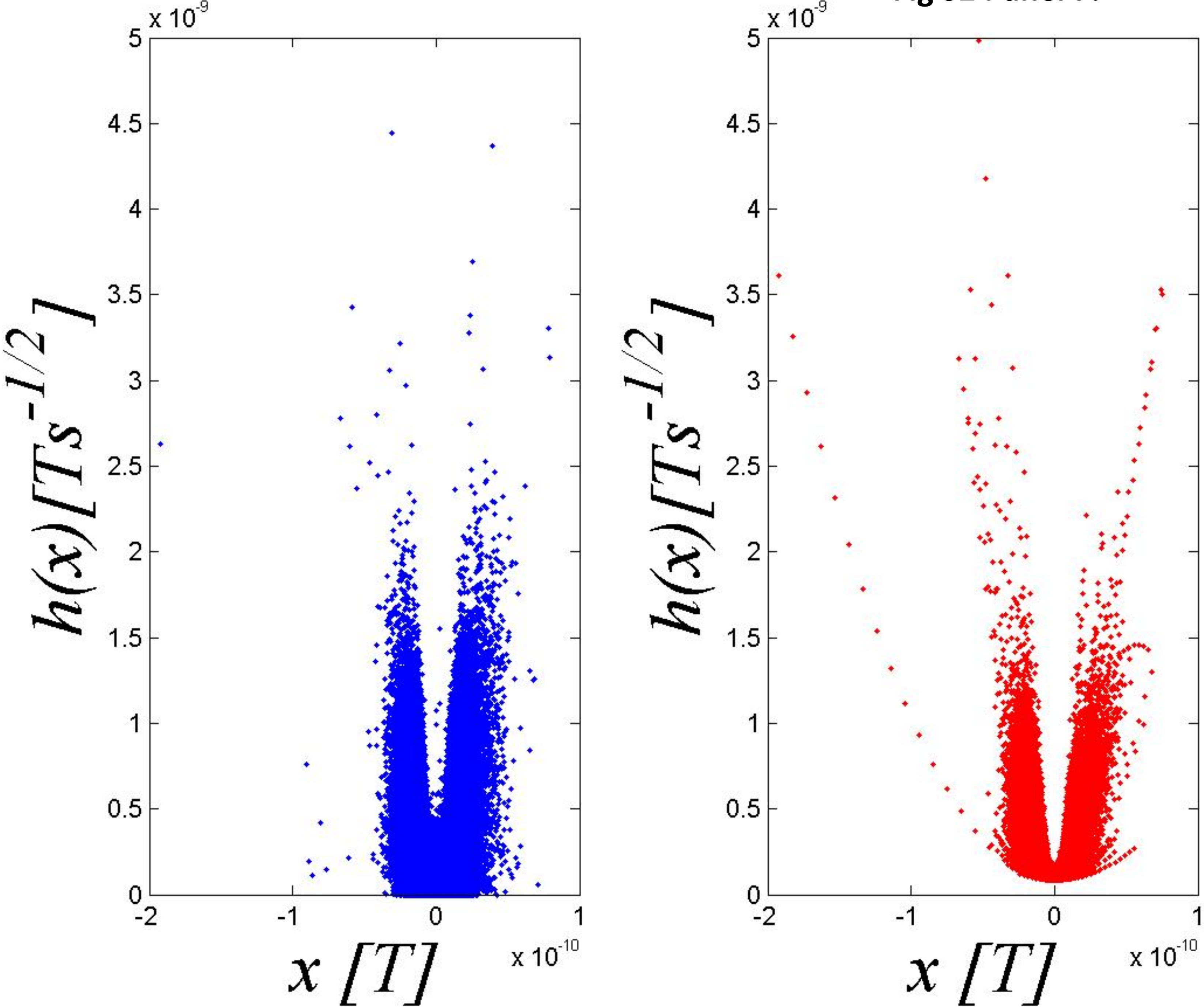

**Fig S2 Panel 8.**

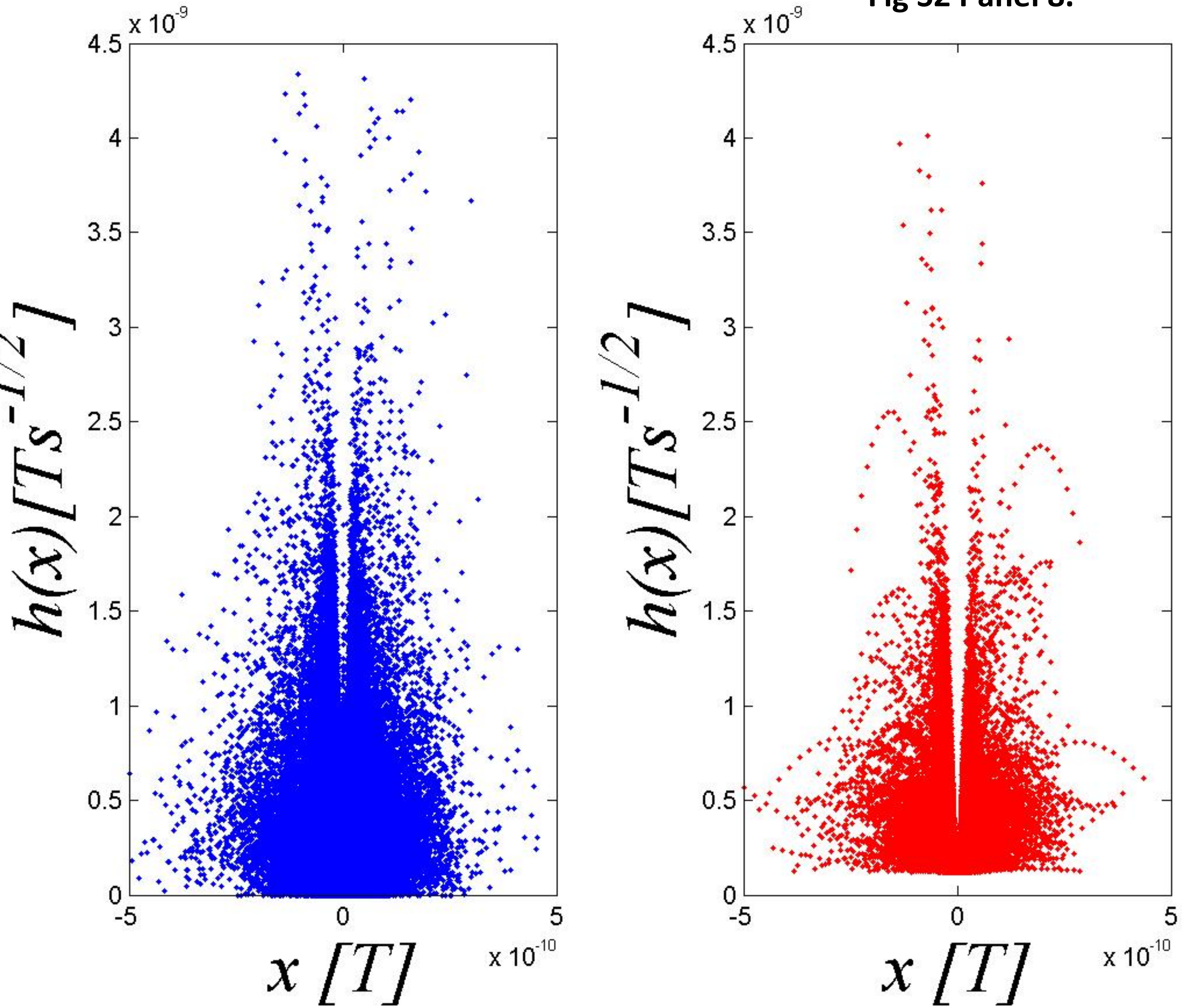

Fig S2 Panel 9.

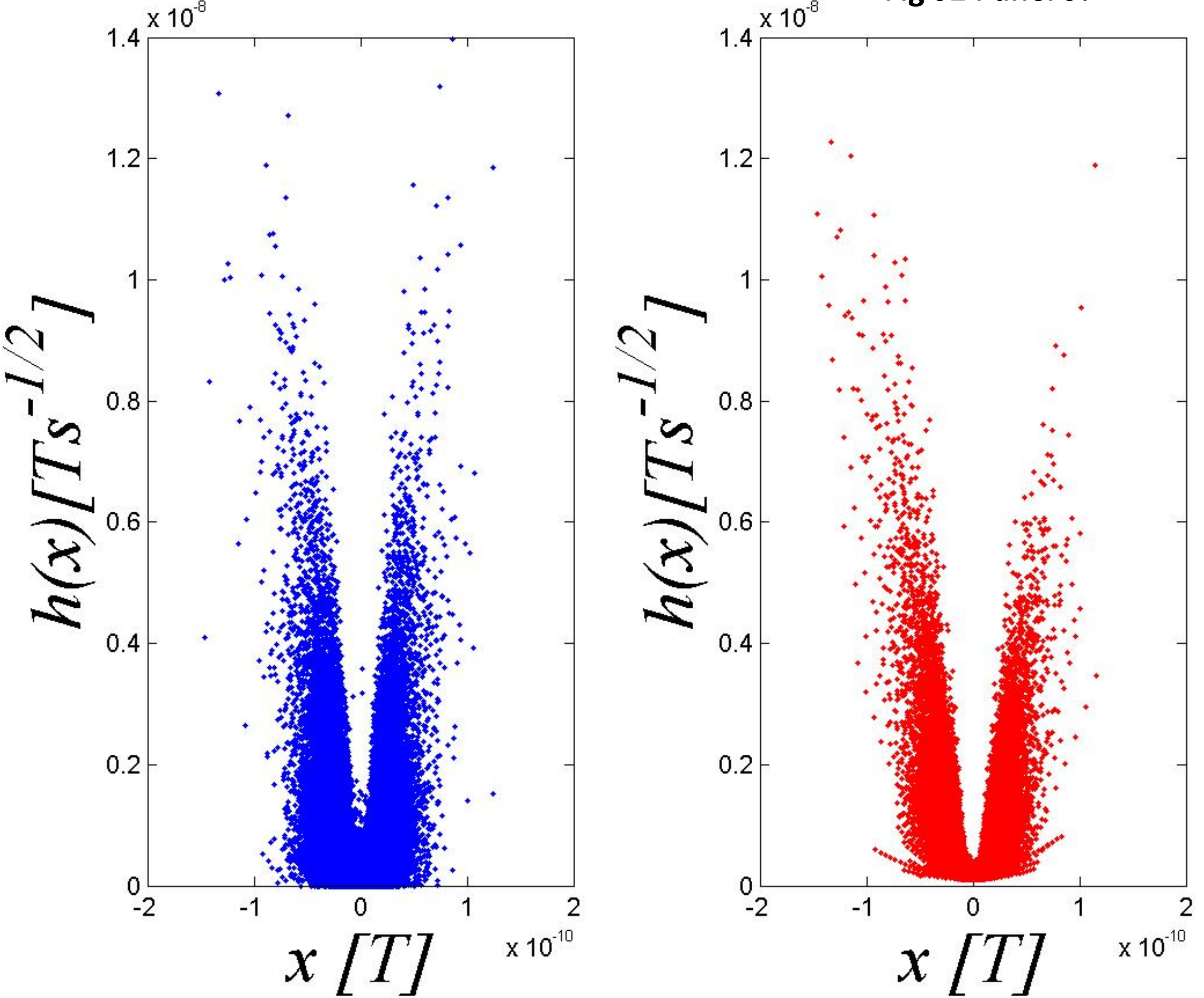

**Fig S2 Panel 10.**

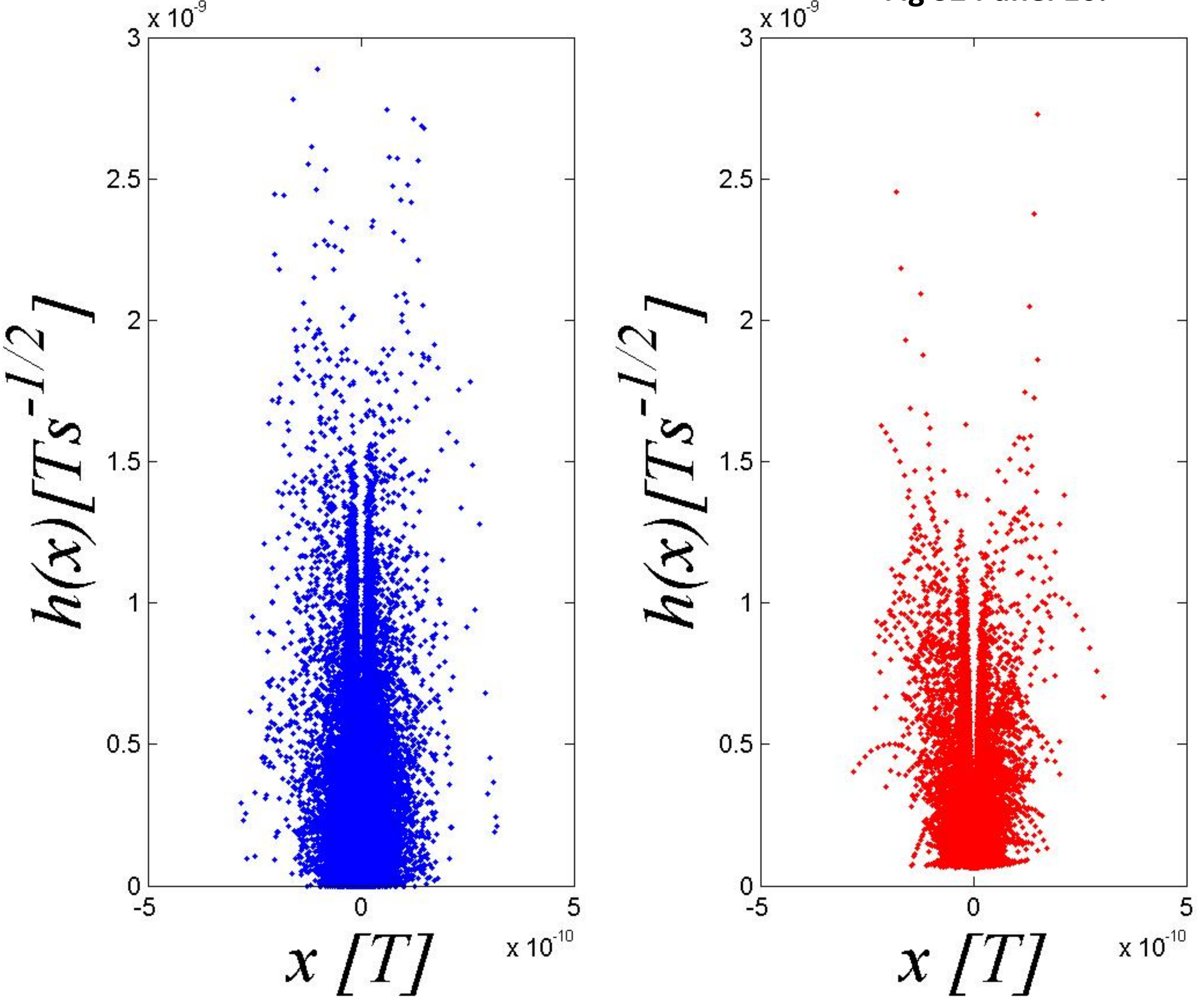

**Fig S2 Panel 11.**

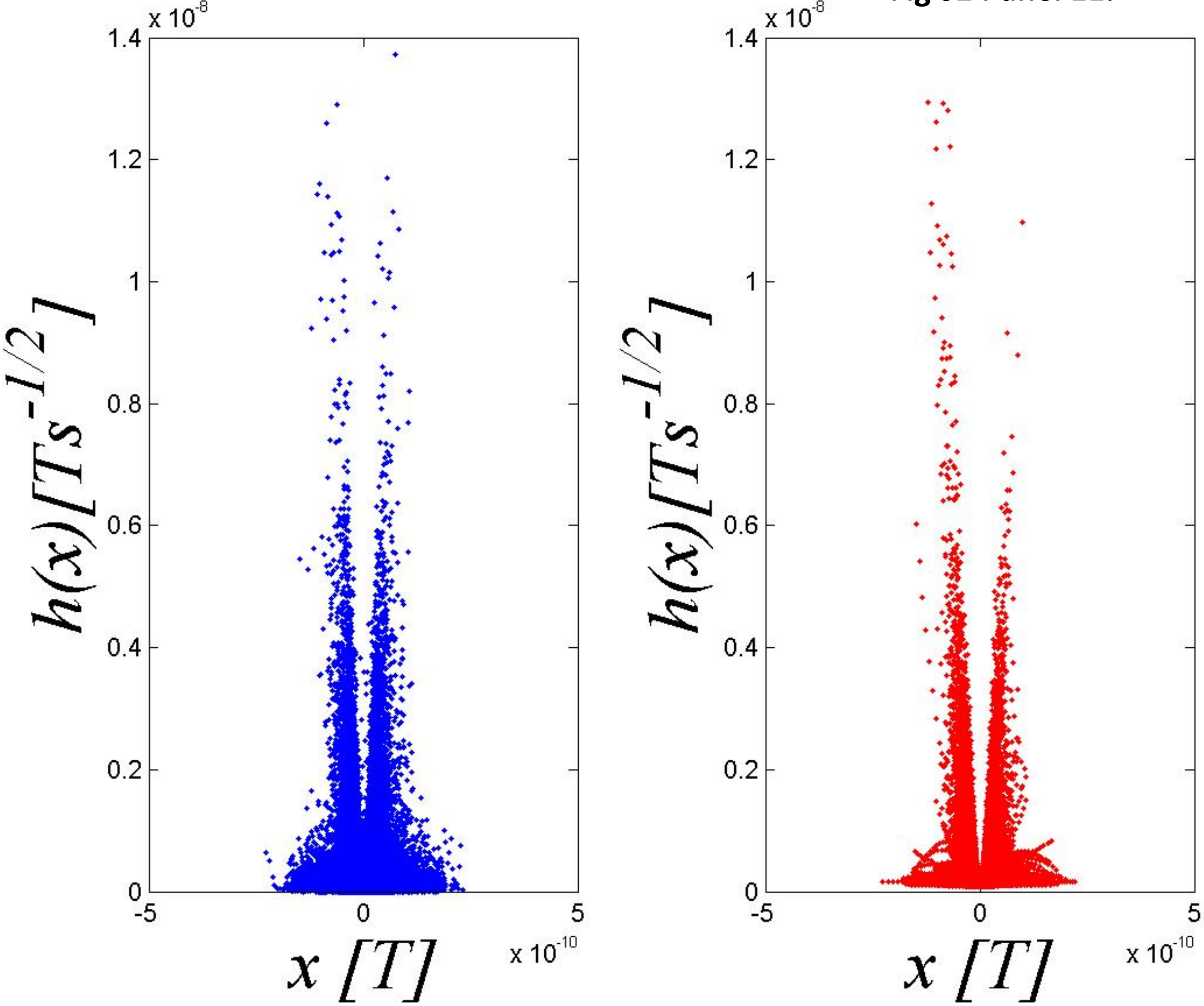

**Fig S2 Panel 12.**

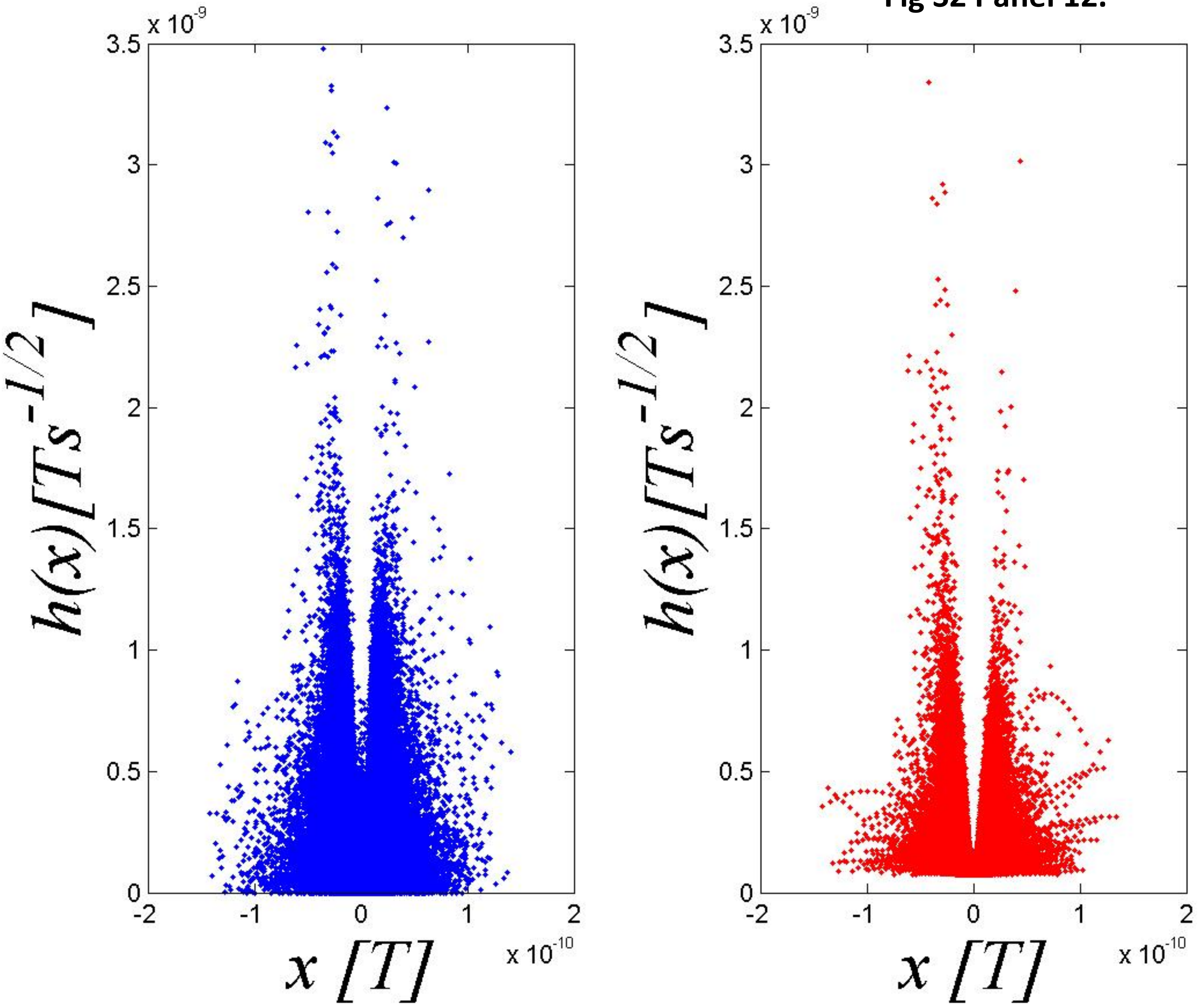

**Fig S2 Panel 13.**

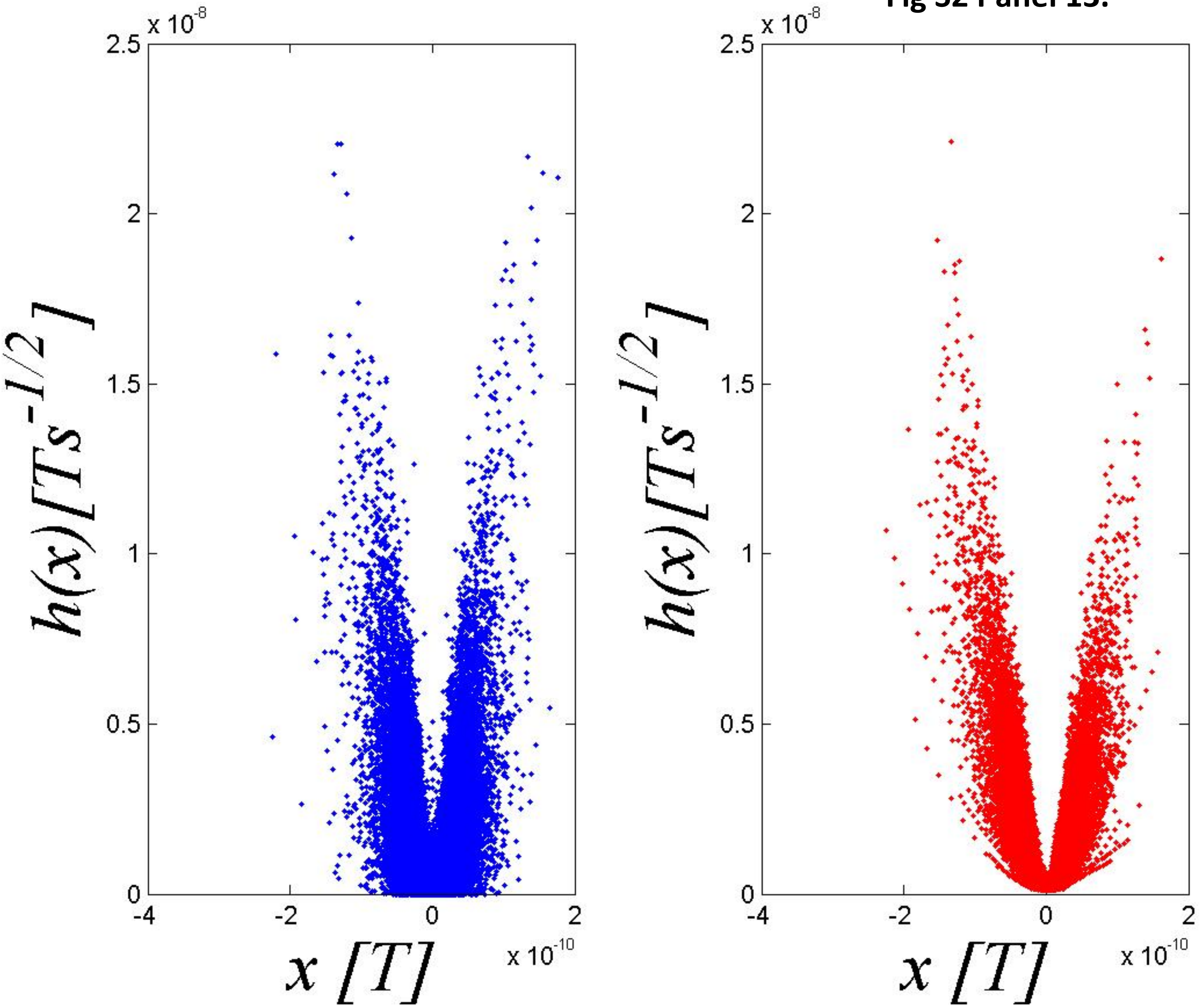

**Fig S2 Panel 14.**

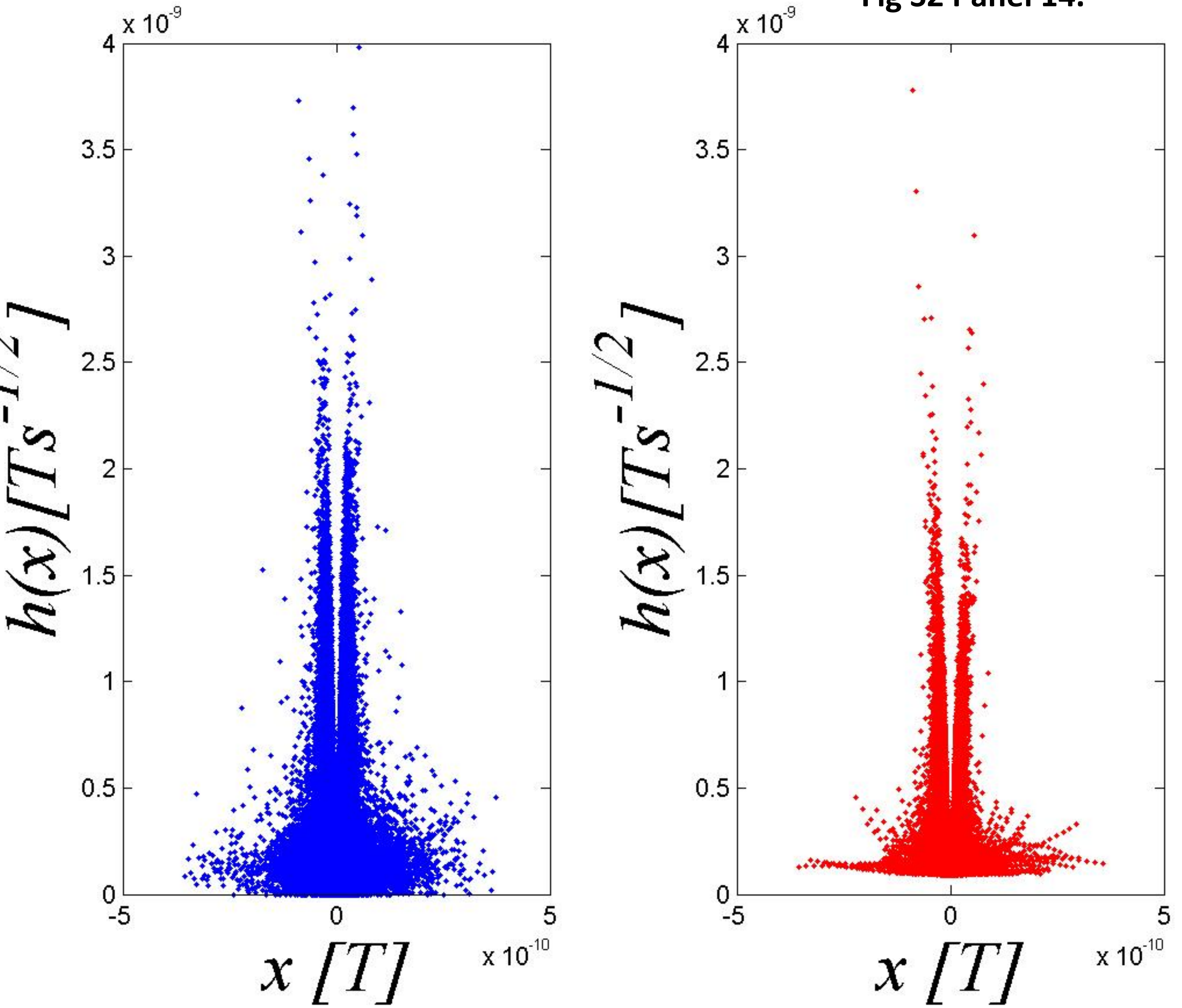

Supplement: Supplementary file 1 — Supplementary Information [file 41598_2019_44197_MOESM1_ESM.pdf]
